# Does *Toxoplasma gondii* infection have a causal impact on human psychopathology? A Mendelian randomization analysis

**DOI:** 10.1371/journal.ppat.1014312

**Published:** 2026-08-04

**Authors:** Jinyong Pang, Matthew J. Valente, Kami Kim, Xiaoming Liu

**Affiliations:** 1 Department of Biostatistics and Data Science, College of Public Health, University of South Florida, Tampa, Florida, United States of America; 2 Department of Internal Medicine, Morsani College of Medicine, University of South Florida, Tampa, Florida, United States of America; 3 Department of Global, Environmental, and Genomic Health Sciences, College of Public Health, University of South Florida, Tampa, Florida, United States of America; University of Geneva Faculty of Medicine: Universite de Geneve Faculte de Medecine, SWITZERLAND

## Abstract

*Toxoplasma gondii* (*T. gondii*) is a prevalent zoonotic parasite that has been implicated in influencing human psychiatric disorders and risk-taking behaviors. Using genome-wide association study (GWAS) data, we selected 25 and 76 single-nucleotide polymorphisms as instrumental variables for anti-*T. gondii* IgG seropositivity and anti-*T. gondii* IgG levels, respectively, and conducted two-sample Mendelian randomization (MR) analyses across 18 GWAS datasets to investigate potential causal effects on addiction, bipolar disorder, obsessive-compulsive disorder, schizophrenia, and risk-taking behavior in European populations. Contrary to previous epidemiological evidence, our MR analyses do not support a significant causal association between *T. gondii* infection and any of the studied psychiatric disorders or risk-taking behavior. Collectively, these results establish that any true causal effect of genetic liability to *T. gondii* infection is likely to be small (OR < 1.18 for schizophrenia, < 1.29 for bipolar disorder) and below the effect sizes typically reported in observational seroepidemiological studies, although small or infection-phase-specific effects cannot be excluded.

## Author summary

*Toxoplasma gondii* is a single-celled parasite found worldwide. Although cats and their relatives are the only hosts in which it reproduces sexually, the parasite can establish a lifelong, dormant infection in essentially any warm-blooded animal it infects—including humans; roughly one in three people carries such a chronic infection. For decades, researchers have reported that people who test positive for past infection appear more likely to develop schizophrenia, bipolar disorder, and other psychiatric conditions. However, these studies cannot tell us whether the parasite actually causes mental illness or whether other factors explain the link. We used a genetics-based approach called Mendelian randomization, which leverages natural variation in people’s DNA to mimic a randomized trial. By examining whether genetic variants that predispose individuals to *Toxoplasma* infection also increase their risk of psychiatric disorders or risk-taking behavior, we can test for a cause-and-effect relationship while largely avoiding the confounding that plagues traditional observational studies. Our analyses found no evidence that genetic susceptibility to *Toxoplasma* infection causes addiction, bipolar disorder, obsessive-compulsive disorder, schizophrenia, or risk-taking behavior. While we cannot rule out small or infection-stage-specific effects, our results suggest that the associations reported in prior studies are unlikely to reflect a strong causal relationship.

## Introduction

Infection by the protozoan parasite *Toxoplasma gondii* (*T. gondii*) affects approximately one-third of the global human population [1]. This intracellular parasite relies on members of the cat family as definitive hosts for sexual reproduction. All warm-blooded animals can potentially serve as intermediate hosts. Uncooked or undercooked food, as well as water and soil, poses high risks for *T. gondii* infection in humans. These sources may be contaminated by oocysts originating from the feces of infected felines. Once *T. gondii* is ingested by an intermediate host, it first establishes infection as rapidly dividing tachyzoites, which subsequently convert into more slowly replicating bradyzoites enclosed within tissue cysts. These tissue cysts persist for the lifetime of the host and may contribute to alterations of host hormones, neurotransmission within the brain, and neuro-inflammation [2], providing a mechanism by which persistent *T. gondii* infection or inflammation induced by chronic infection could impact neuropsychiatric disease and the host’s behavior. *T. gondii* infection of rodent intermediate hosts influences the behavior of the infected mice, increasing the likelihood of being captured by cats and thus favoring reproduction and transmission [3].

The most consistent epidemiological association has been observed between *T. gondii* infection and schizophrenia. An early case-control study [4] reported elevated seroprevalence of anti-*T. gondii* IgG antibodies among individuals with first-episode schizophrenia compared with controls. A hypothesis-generating case-control study among U.S. military personnel demonstrated that serological evidence of *T. gondii* exposure was associated with increased odds of subsequent schizophrenia diagnosis (OR: 1.24–1.50, depending on the model specifications), suggesting that exposure may precede disease onset [5]. However, given the observational design, the residual confounding factors, especially by socioeconomic status, environmental exposures, and comorbid infections, cannot be excluded.

For bipolar disorder, Rensch et al. [6] examined the relationship between *T. gondii* seropositivity, serointensity, and cognitive function in euthymic patients. Although overall cognitive performance did not differ between seropositive and seronegative participants, higher IgG antibody levels were associated with poorer verbal memory, suggesting a potential link between latent *T. gondii* infection and memory deficits, albeit in a limited sample. Evidence linking *T. gondii* infection to obsessive-compulsive disorder is limited and mainly comes from small case-control studies. Miman et al. [7] reported a higher seropositivity rate in obsessive-compulsive disorder patients (47.6%) compared with controls (19%), although sample sizes were modest and confounding factors were not fully controlled.

A growing body of literature has examined associations between *T. gondii* infection and human behavioral phenotypes, including suicidality and risk-taking. In a large population-based cohort of Danish women, *T. gondii* seropositivity was associated with an increased risk of self-directed violence, with hazard ratios ranging approximately from 1.3 to 2.0, and evidence of a dose-response relationship with IgG antibody levels [8]. Flegr et al. [9] reported that individuals with latent toxoplasmosis had a higher risk of traffic accidents compared with uninfected subjects (OR = 2.65, 95% CI: 1.76–4.01), with risk increasing alongside higher anti-Toxoplasma antibody titers.

Consistent with the primary studies detailed above, systematic reviews and meta-analyses have confirmed significant associations between *T. gondii* seropositivity and multiple psychiatric disorders, excluding major depression [10,11], as well as links to risk-taking behaviors including suicidal behavior and risky driving [11,12].

Although accumulating epidemiological evidence has demonstrated an association between *T. gondii* infection and psychiatric disorders or risk-taking behavior using ORs or adjusted ORs, it still remains unknown whether *T. gondii* infection has a causal effect on psychiatric disorders or risk-taking behavior. To answer this question, in this paper, two-sample Mendelian randomization (MR) analyses were performed to estimate the causal association between presumed exposure-outcome pathways and genome-wide association study (GWAS) data utilizing single nucleotide polymorphisms (SNPs) as instrumental variables (IVs) [13]. MR is analogous to a randomized controlled trial (RCT) but uses genetic variants (single nucleotide polymorphisms, SNPs) as natural randomized instruments to allocate exposure status. Because alleles are randomly assorted at conception according to Mendel’s Law of Independent Assortment, the genetic predisposition to an exposure is randomly distributed across the population. This design can mitigate key limitations of traditional observational studies, such as residual confounding and reverse causation, provided the instrumental variable assumptions are satisfied.

In this study, four psychiatric disorders (addiction, bipolar disorder, obsessive-compulsive disorder, and schizophrenia) and risk-taking behavior were adopted as outcomes in MR analysis. By systematically evaluating the observational associations described above using MR, we aim to clarify whether the reported links between *T. gondii* infection and psychiatric disorders represent true causal effects or are products of residual confounding and reverse causation.

## Results

**Fig 1:**
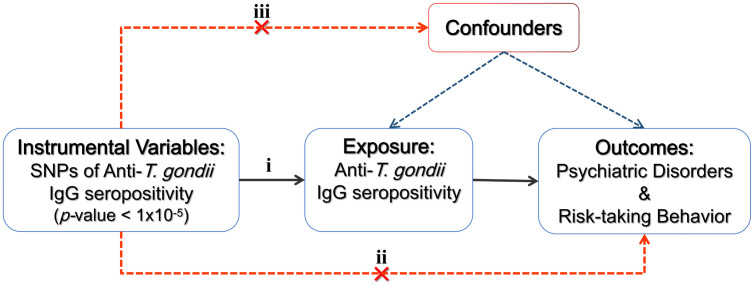
Schematic diagram of the study design and core assumptions of the two-sample Mendelian randomization (MR) analyses. Genetic variants (SNPs) associated with anti–*T. gondii* IgG seropositivity and IgG levels serve as instrumental variables to estimate the causal effect of the exposure (anti–*T. gondii* IgG seropositivity and levels) on the outcomes (psychiatric disorders and risk-taking behavior). Solid black arrows denote the hypothesized causal relationships: (i) the instrumental variables are robustly associated with the exposure (relevance assumption), and the exposure is hypothesized to causally influence the outcomes. Red dashed lines crossed by a red “×” represent the two assumptions that must hold for valid causal inference and are therefore assumed to be absent: (ii) the instrumental variables do not affect the outcomes through any pathway independent of the exposure (exclusion-restriction assumption), and (iii) the instrumental variables are not associated with confounders of the exposure–outcome relationship (independence assumption). Blue dashed arrows indicate potential confounding pathways linking unmeasured confounders to both the exposure and the outcomes.

In the following sections, we first present the major findings of our two-sample MR analyses, then we present each analysis in more detail. Briefly, we utilized genetic variants (SNPs) associated with *T. gondii* infection markers (anti-*T. gondii* IgG seropositivity and anti-*T. gondii* IgG levels) as instrumental variables to estimate the causal effects on psychiatric disorders and risk-taking behavior (Fig 1). For each exposure-outcome pair, we report the primary inverse-variance weighted (IVW) regression results along with sensitivity analyses (MR-Egger, weighted median, and MR-PRESSO) to assess the robustness of our findings.

**Fig 2:**
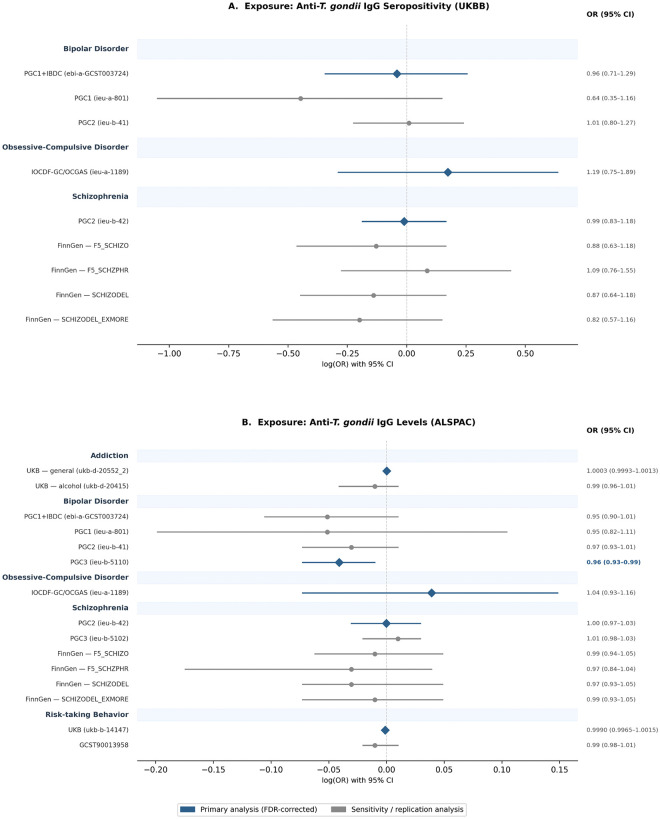
Forest plot of primary IVW Mendelian randomization estimates. Each point represents the IVW causal estimate (log odds ratio) for a primary exposure-outcome pair, with horizontal lines indicating 95% confidence intervals. The dashed vertical line at zero represents no causal effect. Exposure measures include anti-*T. gondii* IgG seropositivity and anti-*T. gondii* IgG levels. Outcomes include addiction, bipolar disorder, obsessive-compulsive disorder, schizophrenia, and risk-taking behavior.

A total of 25 and 76 SNPs were selected as IVs for anti-*T. gondii* IgG Seropositivity and anti-*T. gondii* IgG levels, respectively (S1 and S2 Tables). For both the anti-*T. gondii* IgG measures, across all psychiatric disorders and risk-taking behavior, no statistically significant positive causal effect was found, with the exception of a significant negative association between anti-*T. gondii* IgG levels and bipolar disorder in the PGC3 dataset (OR 0.96, 95% CI 0.93–0.99, FDR-adjusted *p* = 0.043) (Figs 2 and S1–S24 and S12 Table). To assess whether these null findings could reflect insufficient power, we calculated the minimum detectable odds ratio at 80% power (α = 0.05) for each primary analysis. For anti-*T. gondii* IgG seropositivity, the minimum detectable ORs were 1.04 for schizophrenia, 1.08 for bipolar disorder, and 1.14 for OCD. For anti-*T. gondii* IgG levels, the analyses were even more sensitive, with minimum detectable ORs of 1.01 for schizophrenia, bipolar disorder, and risk-taking, 1.06 for OCD, and 1.08 for addiction. For all the tests, no significant heterogeneity or pleiotropy was detected (S1–S24 Figs and S3–S8 Tables). Leave-one-out analyses with sensitivity tests revealed that the effect estimation was not influenced by any dominant SNP (S1–S24 Figs).

Our results showed a significant negative causal association between anti-*T. gondii* IgG levels and bipolar, that is, a higher anti-*T. gondii* IgG level reduces the risk of bipolar (Fig 2 and S12 Table). This contradicts the common hypothesis [14]. However, the significant results were driven by a single dataset ieu-b-5110. We did a colocalization analysis (S11 Table) and the results did not support a shared causal variant between the exposure and outcome at the instrumented loci (PP.H4 < 0.80), suggesting this causal association is likely a false positive. Besides the meta-analysis on anti-*T. gondii* IgG levels with bipolar disorder, the rest of meta-analyses showed there was no significant pooled causal effect of *T. gondii* infection on schizophrenia (95% CIs of ORs all include 1) (S3 and S4 Tables). The complete colocalization analysis (S11 Table) confirmed that no genetic association in our MR analyses has a significantly large posterior probability (PP.H4).

No significant effects were detected by the reverse MR analysis either (S7–S10 Tables).

### Assessing effects of *T. gondii* infection (anti-*T. gondii* IgG seropositivity) on psychiatric disorders

In the four MR analyses with bipolar disorder as the outcome, MR-PRESSO outlier tests revealed the SNP rs147285933 as an outlier of IVs in the GWAS dataset ieu-b-41 (S5 Table), and it was deleted subsequently. IVW regression revealed no significant association between *T. gondii* infection and bipolar disorder (Fig 2 and S12 Table). Heterogeneity tests and MR-Egger intercept tests showed no evidence of heterogeneity or pleiotropy in the four MR analyses (S5 Table). Statistical power and minimum detectable effect sizes for each analysis are reported in S12 Table.

With no outliers detected by MR-PRESSO, the association of *T. gondii* infection with obsessive-compulsive disorder was not statistically significant according to the IVW model (Fig 2 and S12 Table). There was no evidence of heterogeneity or pleiotropy in this two-sample MR analysis (S5 Table).

Among the seven MR analyses investigating the relation between *T. gondii* infection and schizophrenia, IVW regression indicated no statistically significant effects in any of the seven MR analyses (Fig 2 and S12 Table). No heterogeneity and pleiotropy were found in two relevant tests (S5 Table).

Overall, in leave-one-out analyses with sensitivity tests, no SNP exhibited a dominant influence on the estimated effects across each MR analysis involving the outcomes of the four psychiatric disorders (S1–S9 Figs).

### Assessing effects of *T. gondii* infection (anti-*T. gondii* IgG levels) on psychiatric disorders

During two MR analyses with anti-*T. gondii* IgG levels as the exposure, no outlier SNPs were detected through the MR-PRESSO outlier test for the outcome addiction. The association of *T. gondii* infection with addiction did not achieve statistical significance in IVW regression model (Fig 2 and S12 Table). Neither the GWAS dataset ukb-d-20552_2 nor the GWAS dataset ukb-d-20415 exhibited heterogeneity or pleiotropy (S6 Table).

In four MR analyses with bipolar disorder as the outcome, MR-PRESSO outlier tests revealed the SNP rs60385209 as an outlier of IVs in the GWAS dataset ieu-b-41 (S6 Table), and it was deleted subsequently. IVW regression showed no significant positive association between anti-*T. gondii* IgG levels and bipolar disorder (Fig 2 and S12 Table). Notably, one dataset (ieu-b-5110) did suggest a significant negative association between anti-*T. gondii* IgG levels and bipolar disorder. However, in contrast to the common hypothesis that higher anti-*T. gondii* IgG levels are associated with higher bipolar risk [14,15], and the result suggests the opposite. Heterogeneity tests and MR-Egger intercept tests showed no evidence of heterogeneity or pleiotropy in the four MR analyses (S6 Table).

With no outliers detected by MR-PRESSO, the association of anti-*T. gondii* IgG levels with obsessive-compulsive disorder was not statistically significant according to the IVW model (Fig 2 and S12 Table). There was no evidence of heterogeneity or pleiotropy in this two-sample MR analysis (S6 Table).

Among the seven MR analyses investigating the relation between anti-*T. gondii* IgG levels on schizophrenia, two SNPs, rs114230280 and rs1264457, in the GWAS dataset ieu-b-42 were identified as outliers via the MR-PRESSO outlier test. IVW regression indicated no statistically significant causal effects in any of the seven MR analyses (Fig 2 and S12 Table). No heterogeneity and pleiotropy were found in two relevant tests (S6 Table).

Overall, in leave-one-out analyses with sensitivity tests, no SNP exhibited a dominant influence on the estimated effects across each MR analysis involving the outcomes of the four psychiatric disorders (S10–S22 Figs).

### Assessing the effects of *T. gondii* infection (anti-*T. gondii* IgG levels) on risk-taking behavior

Four summarized GWAS datasets on risk-taking behavior were included as the outcome of our study. One outlier, rs77236966, was identified in the GWAS data ebi-a-GCST90013958 based on MR-PRESSO tests (S6 Table). IVW regression yielded nonsignificant estimates for the effect of anti-*T. gondii* IgG levels on risk-taking behavior (Fig 2 and S12 Table). Heterogeneity and MR-Egger intercept tests confirmed the absence of heterogeneity and pleiotropy in all four two-sample MR analyses (S6 Table). Leave-one-out analyses with sensitivity tests revealed that the effect estimation was not influenced by any dominant SNP (S23 and S24 Figs).

### Meta-analysis with multiple two-sample MR studies

To further increase the power to detect the potential causal impact of *T. gondii* infection (anti-*T. gondii* IgG seropositivity & levels), we conducted a meta-analysis for the outcome with multiple independent GWAS datasets in two-sample MR analyses, including bipolar disorder and schizophrenia. Neither group of meta-analyses yielded significant pooled causal effects (S3 and S4 Tables).

Moreover, Cochran’s Q test revealed that none of the four meta-analyses in our study exhibited significant heterogeneity (S3 and S4 Tables). The Higgins I^2^ index statistics in meta-analyses for two-sample MR studies consistently approached 0%, indicating homogeneity among different two-sample MR studies (S3 and S4 Tables). The statistics τ^2^ for each meta-analysis were zero within a tiny standard error, signifying no variability in true effect sizes across different two-sample MR studies (S3 and S4 Tables). It was confirmed through leave-one-out analysis with sensitivity tests that the overall causal effect estimation was not influenced by any dominant two-sample MR analysis (S9 and S10 Tables).

### Reverse MR analyses

We also conducted reverse MR analysis to assess the causal impacts of psychiatric disorders or risk-taking behavior on *T. gondii* infection (anti-*T. gondii* IgG seropositivity & anti-*T. gondii* IgG levels). In the context of homogeneous MR analyses, no significant effects were detected in the reverse path of the MR (S7–S10 Tables).

### Colocalization analysis

To further validate the genetic associations and determine whether a shared biological mechanism links the exposure and outcome traits, we conducted Bayesian colocalization analyses within the genomic regions surrounding our strictly filtered lead SNPs. Utilizing the distinct instrumental variables derived from the two exposure datasets, we systematically evaluated the posterior probabilities for each hypothesis. Our results indicated a lack of robust evidence for colocalization across all tested loci. Specifically, the posterior probability of the exposure and outcome sharing a single causal variant (PP.H4) remained consistently low and did not reach the predefined threshold for strong evidence (PP.H4 < 0.80). These findings demonstrate that, within the examined genomic regions, there are no shared causal genetic signals driving both the exposure and the psychiatric outcomes.

### Multiple testing correction with selection of primary independent analyses

To stringently control the expected proportion of false positives among the declared significant results, we applied the Benjamini-Hochberg False Discovery Rate (FDR) correction [16]. In large-scale Mendelian randomization (MR) studies incorporating multiple iterations of consortia data (PGC1, PGC2, PGC3) and nested sub-phenotypes (FinnGen), blindly applying multiple testing corrections across all available datasets introduces severe conservative bias. Therefore, to define the appropriate denominator for the FDR adjustment, we established a rigorous a priori protocol to select primary independent analyses based on three core criteria, including strict avoidance of sample overlap to prevent weak instrument bias toward observational estimates [17], maximization of statistical power by prioritizing cohorts with the largest effective sample sizes and highest number of confirmed cases [18], and phenotypic independence. Applying this framework, we identified exactly eight independent primary biological hypotheses: three seropositivity tests (bipolar disorder, OCD, schizophrenia) plus five IgG-level tests (addiction, bipolar disorder, OCD, schizophrenia, risk-taking behavior), after excluding two overlapping pairings in which the UK Biobank-derived seropositivity exposure shared 100% sample overlap with UK Biobank-derived outcomes (addiction and risk-taking behavior). Overlapping exposure-outcome pairings (e.g., the UK Biobank-derived exposure against UK Biobank-derived outcomes) were strictly excluded from the primary analyses. Furthermore, when analyzing highly correlated sub-phenotypes, such as the four diagnostic variations of schizophrenia and schizotypal disorders within the FinnGen isolate, or sequentially nested cohorts (PGC iterations), only the single most highly powered and non-overlapping dataset was designated as the primary test. Therefore, the Benjamini-Hochberg FDR correction was applied exclusively to the *p*-values derived from these eight independent primary IVW regression models. An FDR-adjusted *p*-value of less than 0.05 was considered to provide statistically significant evidence of a causal effect. All remaining datasets, including earlier PGC iterations and specific FinnGen diagnostic sub-classifications, were retained strictly as phenotypic sensitivity analyses and independent replication cohorts to assess the robustness of causal directions, and thus did not accrue additional multiple-testing penalties.

## Discussion

This study contributes to the growing body of literature by utilizing a two-sample Mendelian randomization framework to systematically evaluate the potential causal effects of *T. gondii* infection (anti-*T. gondii* IgG seropositivity and levels) on four primary psychiatric disorders and risk-taking behavior. Our genetic analyses do not support a moderate-to-large lifetime causal effect of genetic liability to *T. gondii* infection susceptibility or antibody levels on the studied psychiatric disorders (including addiction, bipolar disorder, obsessive-compulsive disorder, and schizophrenia) or risk-taking behaviors in European populations. However, because genetic instruments proxy lifetime predisposition, they may not capture acute, congenital, or highly localized neuropathological effects of active infection that occur independently of genetic susceptibility. This study provides crucial insights into the relationship between *T. gondii* infection and human psychopathology, emphasizing the need to re-evaluate existing hypotheses in this domain and the necessity of using robust analytical methods such as MR to decipher the intricate interplay between infectious diseases and mental health.

To quantify the range of causal effects that our analyses can exclude, we report the 95% confidence intervals from the primary IVW regression models for each exposure-outcome pair. For anti-*T. gondii* IgG seropositivity, the confidence intervals for schizophrenia (OR 0.83–1.18) rule out causal effects with ORs exceeding 1.18, while for bipolar disorder (OR 0.71–1.29) effects larger than OR 1.29 can be excluded. The confidence interval for OCD remained wider (OR 0.75–1.89), reflecting comparatively lower statistical power for this outcome. For anti-*T. gondii* IgG levels (per-unit increase), the estimates were considerably more precise: schizophrenia (OR 0.98–1.03), addiction (OR 1.00, 95% CI 1.00–1.00), and risk-taking behavior (OR 1.00, 95% CI 1.00–1.00) all showed negligible effect sizes with narrow confidence intervals. The one exception was bipolar disorder (PGC3; OR 0.96, 95% CI 0.93–0.99), which was the only primary analysis reaching nominal and FDR-adjusted significance, although the colocalization analysis did not support a shared causal variant at this locus (PP.H4 < 0.80). Collectively, these confidence intervals establish that, for the psychiatric outcomes examined, any true causal effect of genetic liability to *T. gondii* infection is likely to be small (OR < 1.18 for schizophrenia, < 1.29 for bipolar disorder) and below the effect sizes typically reported in observational seroepidemiological studies.

An important interpretive distinction exists between our two exposure constructs. The binary seropositivity measure (anti-*T. gondii* IgG seropositivity) more directly reflects past infection status, as individuals are seropositive only because they were infected with T. gondii, regardless of whether the associated SNPs act through infection susceptibility or antibody detection thresholds. Consequently, the null results from the seropositivity analyses suggest that genetic liability to *T. gondii* infection is unlikely to have a large causal effect on the studied psychiatric outcomes. By contrast, the continuous IgG levels measure warrants more cautious interpretation. SNPs associated with higher anti-*T. gondii* IgG levels may capture genetic variation in the intensity of the humoral immune response rather than the severity or persistence of infection itself. An individual with genetically vigorous antibody production may exhibit high IgG levels in response to the same parasite burden as someone with lower levels. Thus, the continuous exposure arm may partly test whether genetic propensity to mount a strong antibody response to *T. gondii* is causally related to psychiatric outcomes, which is a related but distinct question from whether infection itself is causal. The convergence of null results across both exposure constructs nevertheless strengthens the overall conclusion, as the two instruments capture complementary aspects of the host-parasite interaction.

The one departure from the otherwise null pattern warrants dedicated consideration. The IVW analysis of anti-*T. gondii* IgG levels against bipolar disorder in the PGC3 cohort (ieu-b-5110) yielded a nominally and FDR-significant negative association (OR 0.96, 95% CI 0.93–0.99; FDR-adjusted *p* = 0.043), suggesting a modest protective effect. However, several lines of evidence argue against interpreting this as a true causal signal. First, this result was driven by a single dataset; the earlier PGC iterations (PGC1, PGC1 + IBDC, PGC2) showed no significant association in the same direction, and the MR-Egger estimate for this cohort was not significant (*p* = 0.165), indicating sensitivity to the choice of pleiotropy model. Second, Bayesian colocalization analysis revealed no evidence of a shared causal variant at the instrumented loci (PP.H4 < 0.80). This pattern is consistent with linkage disequilibrium confounding rather than a shared biological mechanism. Third, the use of relaxed significance thresholds for instrument selection increases susceptibility to the winner’s curse, which could generate spurious associations in the largest outcome dataset. Taken together, while we report this finding transparently, we consider it likely to reflect a statistical artifact rather than genuine immune-mediated neuroprotection.

Our study employed relaxed significance thresholds to select instrumental variants, which warrants consideration. Although sub-genome-wide thresholds may theoretically increase the risk of weak or false-positive instruments, all selected SNPs demonstrated adequate strength (individual F-statistics > 16; mean F-statistics > 19) (S1 and S2 Tables), and multiple sensitivity analyses — including MR-Egger, weighted median, MR-PRESSO, and Cochran’s Q tests — consistently supported the robustness of our findings (S5, S6 and S12 Tables and S1–S24 Figs). Bayesian colocalization analyses yielded no evidence of shared causal variants between the exposure and outcome traits across all tested loci (PP.H4 < 0.80), indicating that the null causal estimates are not attributable to linkage disequilibrium confounding. These colocalization results reinforce the absence of a genetically mediated causal pathway between *T. gondii* infection markers and the investigated psychiatric phenotypes.

Our research is subject to several limitations. First, our MR analyses were conducted exclusively in European populations, limiting generalizability to populations with different genetic architectures or higher *T. gondii* seroprevalence. Second, the use of relaxed significance thresholds (*p* < 1 × 10^−5^ and *p* < 5 × 10^−5^) to select genetic instruments, necessitated by the relatively small exposure GWAS sample sizes, may introduce weak-instrument bias and the winner’s curse, potentially biasing estimates toward the null. Although all selected SNPs exceeded standard instrument-strength thresholds (F-statistics > 16; see preceding paragraph), residual pleiotropy from sub-genome-wide variants cannot be fully excluded. Third, while our primary analyses utilized independent cohorts with zero sample overlap, some secondary analyses involving UK Biobank–derived outcomes may contain partial overlap, which would bias estimates toward positive observational associations; the consistently null results across these analyses argue against overlap-driven false negatives. Fourth, the exposure measures cannot distinguish the type (congenital, acute, chronic, or latent) or duration of *T. gondii* infection, nor do they capture strain-specific virulence. We therefore cannot exclude the possibility that prolonged chronic infection or specific parasite genotypes may have causal effects on psychopathology that population-level genetic instruments fail to detect.

Looking ahead, definitively resolving the question of whether *T. gondii* infection causally affects psychiatric outcomes will require several methodological advances. First, larger *T. gondii* exposure GWAS—ideally with sample sizes exceeding 50,000—are needed to identify genome-wide significant instruments (*p* < 5 × 10–8) and eliminate the reliance on relaxed thresholds. Second, GWAS targeting infection-stage-specific biomarkers (e.g., distinguishing acute IgM responses from chronic IgG persistence, or quantifying brain cyst burden via novel imaging or cerebrospinal fluid markers) would enable MR analyses that address the biological heterogeneity of *T. gondii* infection. Third, MR within seropositive cohorts—examining whether antibody intensity or strain-specific responses predict psychiatric outcomes among those already infected—could isolate dose-response effects that population-level MR cannot detect. Finally, multi-ancestry MR studies incorporating non-European populations with higher *T. gondii* seroprevalence would increase generalizability and statistical power. Until these resources become available, our findings establish an important quantitative constraint: at the level of genetic liability currently measurable, *T. gondii* infection does not confer a moderate-to-large causal effect on the psychiatric outcomes examined.

To place our findings in a broader clinical context, we chose to focus on four major psychiatric disorders (addiction, bipolar disorder, OCD, and schizophrenia) and risk-taking behavior because of their strong and consistent observational associations with *T. gondii* in previous literature. Other mental disorders, such as Major Depressive Disorder (MDD) or Generalized Anxiety Disorder (GAD), were not included because the observational evidence linking these conditions to *T. gondii* is considerably weaker and less consistent than for the four selected disorders. While some individual studies have reported modest associations with depression, large-scale meta-analyses (e.g., Sutterland et al.) [19] have generally found no significant overall association with *T. gondii* seropositivity, making MDD and GAD lower-priority targets for causal exploration given the limited instrument strength available for this study.

## Materials & methods

### Study design

According to Mendel’s law of independent assortment, parental alleles (gene variants) for different traits segregate, or assort, into gametes independently of one another during gamete formation. This process of Mendelian randomization is analogous to the randomization employed in randomized controlled trials [20]. This study leveraged SNPs in GWAS datasets as instrumental variables (IVs) to explore the potential causal association between the exposure of interest and the outcome. The entire study design for the two-sample MR analysis was developed, as illustrated in Fig 1. Toxoplasmosis infection served as the exposure variable, while the occurrence of four psychiatric disorders and one risk-taking behavior was considered outcome variables.

### Data resource

Genome-wide association study summary-level statistics for two different measures of Toxoplasmosis infection (anti-*T. gondii* IgG seropositivity, and anti-*T. gondii* IgG levels), four types of psychiatric disorders (addiction, bipolar disorders, obsessive-compulsive disorder, and schizophrenia) and risk-taking behavior were obtained from the UK Biobank (UKB) (https://biobank.ctsu.ox.ac.uk/), the Integrative Epidemiology Unit OpenGWAS project (IEU) (https://gwas.mrcieu.ac.uk/), the Finnish Genomics project (FinnGen) (https://public-metaresults-fg-ukbb.finngen.fi/) and the Psychiatric Genomics Consortium (PGC) (https://www.med.unc.edu/pgc/). To avoid the bias of population heterogeneity, all participants in the summarized GWAS data were solely from European populations. A total of 18 summary-level GWAS datasets were utilized in our MR analyses (Table 1).

**Table 1:** Summary of GWAS datasets on exposure and outcomes.

GWAS ID	Traits	Consortium	Case	Control	Sample size	PubMed ID
Exposure: Toxoplasmosis Infection						
ebi-a-GCST90006925	Anti-*T. gondii* IgG Seropositivity	UK Biobank	1123	7612	8735	33204752
ieu-b-4910	Anti-*T. gondii* IgG Levels	ALSPAC	–	–	5010	34804013
Outcome: Addiction						
ukb-d-20552_2	Behavioral & miscellaneous addictions	UK Biobank	1216	115530	116746	32026800
ukb-d-20415	Ongoing addiction to alcohol	UK Biobank	1184	1555	2739	32026800
Outcome: Bipolar Disorder						
ebi-a-GCST003724	Bipolar Disorder	IBDC/PGC1	9747	14278	24025	27329760
ieu-a-801	Bipolar Disorder	PGC1	7481	9250	16731	21926972
ieu-b-41	Bipolar Disorder	PGC2	20352	30381	50733	31043756
ieu-b-5110	Bipolar Disorder	PGC3	41917	371549	413466	34002096
Outcome: Obsessive Compulsive Disorder						
ieu-a-1189	Obsessive Compulsive Disorder	IOCDF-GC/OCGAS	2688	7037	9725	28761083
Outcome: Schizophrenia						
ieu-b-42	Schizophrenia	PGC2	34241	45604	79845	25056061
ieu-b-5102	Schizophrenia	PGC3	52017	75889	127906	35396580
finn-b-F5_SCHIZO	Schizophrenia, schizotypal & delusional disorders	FinnGen	10118	208674	218792	36653562
finn-b-F5_SCHZPHR	Schizophrenia	FinnGen	5562	208674	214236	36653562
finn-b-KRA_PSY_SCHIZODEL	Schizotypal & Delusional	FinnGen	10091	208701	218792	36653562
finn-b-KRA_PSY_SCHIZODEL_EXMORE	Schizotypal & Delusional (Strict)	FinnGen	10091	166584	176675	36653562
Outcome: Risk-taking Behavior						
ukb-b-14147	Risk-taking Behavior	UK Biobank	117515	328764	446279	32026800
ebi-a-GCST90013958	Self-reported risk-taking behavior	UK Biobank	113882	322354	436236	30271922

Note: ALSPAC: Avon Longitudinal Study of Parents and Children; IDBC: International Bipolar Disorder Consortium; PGC: Psychiatric Genomics Consortium; IOCDF-GC: International Obsessive Compulsive Disorder Foundation Genetics Collaborative; OCGAS: Obsessive Compulsive Disorder Collaborative Genetics Association Study; FinnGen: The Finnish Genomics Initiative.

To ensure robust genetic instrument selection, we utilized two distinct exposure datasets representing different constructs of *Toxoplasma gondii* infection: (1) Anti-*T. gondii* IgG seropositivity (ebi-a-GCST90006925): This dataset represents a binary susceptibility trait (N = 8,735; 1,123 cases and 7,612 controls) derived from a sub-cohort of the UK Biobank (UKBB) [21]. Seropositivity was defined as a binary trait based on the manufacturer’s recommended cut-off (e.g., > 10 IU/mL for *T. gondii*). (2) Anti-*T. gondii* IgG levels (ieu-b-4910): This dataset represents a continuous antibody level trait (N = 5,010) from the Avon Longitudinal Study of Parents and Children (ALSPAC) birth cohort, reflecting the intensity of the humoral immune response among exposed individuals. Our outcome datasets incorporated large-scale consortia and national registry cohorts with rigorous case definitions: Addiction was sourced from the UK Biobank and PGC, where phenotypes (alcohol, nicotine, and cannabis dependence) were defined using DSM-IV and ICD-10 criteria or validated self-report clinical questionnaires. Bipolar Disorder was sourced from the PGC3 consortium (N = 413,466; 41,917 cases and 371,549 controls), with cases diagnosed according to DSM-IV or ICD-10 criteria across multiple clinical sites. Obsessive-Compulsive Disorder (OCD) was sourced from the PGC (ieu-a-1189; N = 9,725; 2,688 cases and 7,037 controls), utilizing DSM-IV clinical case definitions. Schizophrenia was sourced from both the PGC3 consortium (N = 161,405; 76,755 cases and 84,650 controls) and the FinnGen R9 cohort, where cases were identified via national hospital discharge and death registries using ICD-9 and ICD-10 codes (F20 schizophrenia spectrum disorders).

### Phenotype and cohort

#### Exposure: Toxoplasmosis.

To comprehensively instrument *Toxoplasma gondii* infection, we utilized two distinct exposure datasets. The first dataset was derived from a sub-cohort of the UK Biobank (UKBB; ebi-a-GCST90006925), which assessed exposure as a binary trait. According to the original GWAS, “seropositivity was defined as a binary trait based on the manufacturer’s recommended cut-off (e.g., >10 IU/mL for *T. gondii*)” [21]. To minimize cohort-specific biases and evaluate immune response intensity, a second, independent dataset was sourced from the Avon Longitudinal Study of Parents and Children (ALSPAC; ieu-b-4910), restricted to individuals of European ancestry. As described in the study, “children from the longitudinal birth cohort... had 14 antibodies measured in plasma at age 7... including *Toxoplasma gondii*, and SAG1 protein domain, a surface antigen of *Toxoplasma gondii* measured for greater precision” [22].

#### Outcome: Addiction.

Summary statistics for addiction phenotypes were exclusively sourced from the UK Biobank cohort. The primary trait, assessing general behavioural and miscellaneous addictions (ukb-d-20552_2), was defined by “self-reported addiction, or dependency, to any substance or activity (UKB Data-Field 20552)” [23]. A secondary trait focusing specifically on alcohol use (ukb-d-20415) was similarly ascertained based on “ongoing addiction to alcohol, defined by participants’ self-report (UKB Data-Field 20415)” [23].

#### Outcome: Bipolar disorder.

Our analyses for bipolar disorder incorporated multiple large-scale GWAS datasets reflecting the iterative expansions of the Psychiatric Genomics Consortium (PGC). Initial assessments utilized the PGC1 discovery sample (ieu-a-801), comprising samples “collected from 11 international sites and that were of European ancestry,” where “cases met international consensus criteria (DSM-IV) for bipolar disorder (type I or type II), ascertained by structured clinical interview or review of medical records” [24]. This was supplemented by a meta-analysis combining the PGC1 cohort with the International Bipolar Disorder Consortium (IBDC) (ebi-a-GCST003724) [25]. We also utilized the expanded PGC2 dataset (ieu-b-41), consisting of “32 cohorts from Europe, North America and Australia,” where “cases were required to meet international consensus criteria (DSM-IV, ICD-9 or ICD-10) for a lifetime diagnosis of bipolar disorder (BD), including BD type I... BD type II... schizoaffective disorder bipolar type (SAB) or BD not otherwise specified” [26]. Finally, the massive PGC3 cohort (ieu-b-5110) was evaluated. The PGC3 sample size expansion was “driven by the inclusion of large population-based cohorts, such as the UK Biobank and iPSYCH” [27]. Within this specific UKBB sub-cohort of PGC3, “cases were identified via a combination of self-report (Field 20126), hospital register data (ICD-10 codes F30-F31), and the mental health online questionnaire” [27].

#### Outcome: Obsessive compulsive disorder (OCD).

Genetic association data for obsessive-compulsive disorder (OCD; ieu-a-1189) were obtained from a comprehensive meta-analysis conducted by the International Obsessive Compulsive Disorder Foundation Genetics Collaborative (IOCDF-GC) and the OCD Collaborative Genetics Association Study (OCGAS). The dataset comprised individuals of European ancestry whose samples were “collected through various specialist OCD clinics or research centers in the United States, Canada, and Europe” [28]. Phenotypic stringency was rigorously maintained, as “all cases met DSM-IV criteria for OCD,” and diagnoses “were confirmed using the Structured Clinical Interview for DSM-IV (SCID-I or SCID-IV) or, for pediatric cases, the Kiddie-Schedule for Affective Disorders and Schizophrenia (K-SADS)” [28].

#### Outcome: Schizophrenia.

For schizophrenia, we extracted summary statistics from both the PGC and FinnGen consortia to enable robust meta-analytical validation. From the PGC, we utilized the PGC2 cohort (ieu-b-42), derived from “49 European ancestry cohorts,” wherein “cases were required to meet international consensus criteria (DSM-IV or ICD-10) for a lifetime diagnosis of schizophrenia or schizoaffective disorder, as confirmed by structured clinical interview or review of medical records” [29]. Furthermore, we analyzed the broader PGC3 cohort (ieu-b-5102), which combined “90 cohorts... predominantly of European ancestry,” noting that “for population-based samples like UK Biobank and iPSYCH, cases were identified via national health registers using ICD codes (F20 for schizophrenia)” [30]. To conduct independent meta-analyses free of UKBB overlap, we systematically incorporated four distinct phenotypic definitions from the FinnGen (R10) study, which “reports results from 342,499 individuals from 13 biobanks in Finland” [31]. These FinnGen traits ranged from narrow definitions, such as strictly defined schizophrenia (finn-b-F5_SCHZPHR; “diagnosis of Schizophrenia based on ICD-10 code F20 from national health registers”), to broader diagnostic categories including schizophrenia, schizotypal and delusional disorders (finn-b-F5_SCHIZO; ICD-10 codes F20-F22), general schizotypal and delusional disorders (finn-b-KRA_PSY_SCHIZODEL; “ICD-10 codes: F20-F29”), and a strict variant of schizotypal and delusional disorders with “more strict exclusion of related comorbidities” (finn-b-KRA_PSY_SCHIZODEL_EXMORE).

#### Outcome: Risk-taking.

Risk-taking behaviour phenotypes were sourced from the UK Biobank. The GWAS datasets for these traits (ukb-b-14147 and ebi-a-GCST90013958) utilized identical phenotypic definitions derived from self-reported questionnaire data, ‘Would you describe yourself as someone who takes risks?’ (UKB Field 2040). Consistent with the original study methodologies, risk-taking propensity was modeled based on the “binary response: ‘Would you describe yourself as someone who takes risks?’” [23,32].

#### Sample overlap and bias assessment.

In two-sample Mendelian randomization (MR) analyses, substantial sample overlaps between the exposure and outcome datasets can introduce bias in the causal estimates, shifting them toward observational associations, particularly when genetic instruments are weak. Based on the data sources utilized in this study, the potential for cohort overlap was carefully evaluated across all exposure-outcome pairings. A significant degree of sample overlap is present if utilizing the Toxoplasmosis infection exposure dataset derived from the UK Biobank (UKBB; ebi-a-GCST90006925) to assess outcomes of Addiction (ukb-d-20552_2; ukb-d-20415) and Risk-taking Behavior (ukb-b-14147; ebi-a-GCST90013958). Since both the exposure and these specific outcome GWAS summary statistics originate entirely from the UKBB cohort, this creates a 100% nested or completely overlapping sample scenario. Consequently, standard two-sample MR estimators may be biased, necessitating cautious interpretation or the application of one-sample MR frameworks and robust weak-instrument-resistant methods for these specific traits. Potential partial overlap exists when investigating the outcomes of bipolar disorder and schizophrenia using the latest Psychiatric Genomics Consortium (PGC3) datasets (ieu-b-5110 and ieu-b-5102, respectively). Recent large-scale PGC3 meta-analyses frequently incorporate UKBB participants as one of their sub-cohorts. Therefore, using the UKBB-derived toxoplasmosis exposure against PGC3 outcomes may involve an undetermined proportion of sample overlap. To ensure strict adherence to the core assumptions of two-sample MR and to mitigate the risk of sample overlap bias, several exposure-outcome pairings in our study were strategically derived from completely independent populations. For instance, the ALSPAC cohort (Anti-*T. gondii* IgG Levels; ieu-b-4910) serves as an independent exposure dataset that does not overlap with the UK Biobank (UKBB), FinnGen, or the majority of Psychiatric Genomics Consortium (PGC) cohorts, thereby providing a valuable replication avenue free from overlap bias. Similarly, because the FinnGen cohorts for schizophrenia and schizotypal disorders (e.g., finn-b-F5_SCHIZO, finn-b-F5_SCHZPHR) consist exclusively of the Finnish population, pairing UKBB or ALSPAC exposure datasets with FinnGen outcomes guarantees completely non-overlapping samples. Furthermore, we leveraged earlier iterations of specialized consortia, such as IOCDF-GC/OCGAS (for obsessive-compulsive disorder) and earlier PGC releases, to maintain sample independence. It is crucial to note the successive, nested structure of the PGC datasets: PGC2 encompasses the original PGC1 cohorts, and the subsequent PGC3 expands upon PGC2 by integrating additional large-scale biobanks, frequently including the UKBB. Because the PGC1 and PGC2 datasets (utilized for bipolar disorder and schizophrenia) predated the incorporation of UKBB data, employing these earlier iterations ensures that their pairings with UKBB-derived exposures remain strictly independent, effectively bypassing the overlap issues associated with the broader PGC3 meta-analyses.

### Instrumental variable selection

The selection of IVs is a crucial step before conducting two-sample MR analyses. This is because SNPs, as exposure proxies, must ensure valid causal inference. Therefore, the SNPs included in this research should satisfy three core assumptions for IVs: i) The relevance assumption: the single nucleotide polymorphisms (SNPs) used as instrumental variables (IV) should be strongly correlated with the exposure of interest; ii) The exclusion-restriction assumption: the IVs should exclusively influence the outcome through the exposure and not exhibit pleiotropic effects; and iii) The independence assumption: the IVs should not be associated with any confounding factors related to the exposure-outcome relationship. First, the SNPs strongly associated with anti-*T. gondii* IgG seropositivity and anti-*T. gondii* IgG levels were extracted at a genome-wide significance threshold of *p* < 1 × 10^-5^ and *p* < 5 × 10^-5^, respectively, to ensure that the selected IVs fulfilled the assumptions. The relaxation of the genome-wide significance threshold (5 × 10^-8^) was implemented to ensure an adequate sample size of SNPs for solid two-sample MR analyses [33]. Second, applying linkage disequilibrium (LD) clumping with PLINK (v1.9) [34] ensured the selection of independent SNPs (LD threshold: LD *r*^2^ < 0.001, LD region width > 10,000 kb), in which sequencing data from the 1000 Genomes Project Phase 3 were used to estimate LD. Then, the *F*-statistics, defined as $\frac{N-k-\text{1}}{k}\;\cdot \;\frac{R^{\text{2}}}{\text{1}-R^{\text{2}}}\;$ (*N*: sample size of participants; *k*: number of independent SNPs; $R^{\text{2}}$: variance in exposure explained by SNPs) [35], was calculated to assess the strength of each filtered SNP. SNPs with *F*-statistics less than 10 were considered weak IVs and were excluded.

To eliminate the impact of horizontal pleiotropy bias and ensure adherence to the second assumption, we rigorously validated all screened independent SNPs by using the PhenoScanner online database of genotype-phenotype associations (http://www.phenoscanner.medschl.cam.ac.uk) (date checked Dec. 2023). This verification process involved assessing whether the selected SNPs were associated with secondary phenotypes, considering a stringent significance threshold (*p* < 5 × 10^-8^). Ultimately, a set of 25 and 76 genome-wide significant SNPs serving as IVs strongly associated with the exposure to *T. gondii* infection was selected carefully through screening of the summary-level GWAS dataset ebi-a-GCST90006925 and ieu-b-4910 (see S1 and S2 Tables). Before conducting two-sample Mendelian randomization, palindromic SNPs in two sets of IVs with minor allele frequencies (MAFs) exceeding 0.42 or SNPs with allele mismatches were excluded during the data harmonization process.

### Statistical analysis

Two-sample MR analyses, investigating causal associations between an exposure (risk factor) and an outcome using genetic data from two independent samples or datasets, were subsequently conducted with two R packages, “TwoSampleMR” [36] and “MR-PRESSO” [37]. The priori statistical power for MR analyses was calculated via the Shiny web application (https://sb452.shinyapps.io/power/)[18].

To evaluate the causal effect of *T. gondii* infection (anti-*T. gondii* IgG seropositivity and anti-*T. gondii* IgG levels) on four different psychiatric disorders and risk-taking behavior, two-sample MR analyses were implemented by using the inverse variance weighting (IVW) approach [38], the MR-Egger regression approach [39], the MR-Pleiotropy RESidual Sum and Outlier (MR-PRESSO) approach [37] and the weighted median approach [40]. The various IV regression methods used in two-sample MR analysis involve distinct model assumptions, and our study leveraged different approaches, each of which plays a unique role based on these assumptions.

The IVW regression method provided robust and accurate estimates of causal effects under the assumption of no horizontal pleiotropy. However, the MR-Egger method and the weighted median method were used to conduct MR analysis, assuming the presence of pleiotropy in >50% of the SNPs[39] and <50% of the SNPs[40], respectively. The MR-PRESSO method not only provides a corrected estimate of causal effects on pleiotropy but also allows statistical tests to recognize and delete outlier and pleiotropic SNPs in MR analyses under the assumption of pleiotropy [37]. Consequently, the primary MR analyses relied on the IVW method, provided that no horizontal pleiotropy was detected in the sensitivity analyses. In the presence of horizontal pleiotropy, the MR-Egger regression method can provide more valid Mendelian randomization estimates than the IVW method [41]. When heterogeneity is identified among the IVs without the presence of pleiotropy, employing the weighted median method can provide robust estimates in two-sample MR analysis [40].

To assess the reliability of the results of a two-sample MR analysis, it is crucial to conduct a comprehensive sensitivity analysis, which helps to check and address underlying horizontal pleiotropy and heterogeneity. The MR-Egger intercept test can detect potential violations of the second assumption regarding horizontal pleiotropy with a *p-value* less than 0.05. Furthermore, the MR-PRESSO method can reveal the existence of overall horizontal pleiotropic effects by implementing global tests and checking for horizontal pleiotropic outliers by performing outlier tests. After excluding the outlier SNP with an individual *p-value* less than 0.05 in the outlier test, the MR-PRESSO global and outlier tests were repeated for the remaining SNPs until the global test yielded statistical nonsignificance (*p* > 0.05). The subsequent two-sample MR analysis was carried out on the remaining SNPs. Cochran’s *Q* test detected heterogeneity among SNPs in the IVW regression, where a Q *p-*value above 0.05 indicated that the null hypothesis of no heterogeneity could not be rejected [42]. Leave-one-out analyses with sensitivity tests were employed as a useful tool for detecting genetic variants that dominate the causal effect estimate. The removal of such SNPs can enhance the robustness of the findings in two-sample MR analyses. The reverse two-sample MR analyses were performed following the same procedure as those described above.

To maximize statistical power while rigorously mitigating the risk of Type-I error inflation driven by sample overlap, we implemented a dynamic MR meta-analysis strategy tailored to the independence of the exposure source. For analyses utilizing the UK Biobank (UKBB)-derived exposure (ebi-a-GCST90006925), we deliberately excluded the latest Psychiatric Genomics Consortium cohort (PGC3) due to its extensive incorporation of UKBB participants. Instead, we pooled the MR estimates from the earlier, independent PGC2 cohort (ieu-b-42) and the completely non-overlapping FinnGen cohorts. Conversely, when employing the ALSPAC birth cohort as the exposure (ieu-b-4910), the sample overlap constraint was inherently resolved. This allowed us to safely leverage the largest available dataset, PGC3 (ieu-b-5102), meta-analyzing its results with FinnGen to achieve maximum statistical power. Furthermore, to ensure the reliability of our findings across varying diagnostic boundaries, each meta-analysis pipeline was systematically iterated across four distinct definitions of schizophrenia and schizotypal disorders within the FinnGen consortium (ranging from strict to broad criteria). This cohort-aware grouping strategy ensured that all combined causal estimates were strictly derived from independent populations, yielding robust and unbiased evidence. The pooled estimate of causality was derived through meta-analysis using the R package “metafor” [43]. Cochran’s *Q* test and Higgins *I*^*2*^ index statistics were employed to assess heterogeneity among multiple two-sample MR analyses for each outcome. If the *p-*value in the heterogeneity test exceeded 0.05 and the *I*^2^ index was less than 50%, suggesting homogeneity among two-sample MR analyses, the fixed-effects model was deemed appropriate. The fixed-effects model assumes that effect sizes are consistent across studies and estimates a weighted average of their true values [44]. Conversely, the random effects model was considered more suitable if heterogeneity was detected among two-sample MR analyses (*p-*value < 0.05 or *I*^2^ index statistics ≥ 50%). The random effects model assumes that the study effects are variable and that the studies represent a random sample from a larger population of similar studies [44–46]. The statistics tau-square (τ^2^), an estimate of the between-study variance, was also reported in our meta-analysis, representing the degree of variability in true effect sizes across studies beyond what would be expected owing to random sampling error alone. The greater the value of τ^2,^ the more between-study heterogeneity in the true effect sizes across different two-sample MR studies. Furthermore, Egger’s test was used to evaluate publication bias. A sensitivity test and leave-one-out analysis were used in the meta-analysis to assess the overall results’ robustness and investigate the influence of individual two-sample MR analysis on the summarized causal estimate. These analyses helped identify whether specific studies heavily influence the findings and whether the results are consistent across different GWAS datasets within a given exposure-outcome pathway with causality.

To stringently control the expected proportion of false positives among the declared significant results, we applied the Benjamini-Hochberg False Discovery Rate (FDR) correction [16]. To define the appropriate denominator for the FDR adjustment, we established a rigorous a priori protocol to select primary independent analyses based on three core criteria: strict avoidance of sample overlap, maximization of statistical power by prioritizing cohorts with the largest effective sample sizes, and phenotypic independence. Applying this framework, we identified exactly eight independent primary biological hypotheses (three seropositivity tests (bipolar disorder, OCD, schizophrenia) plus five IgG-level tests (addiction, bipolar disorder, OCD, schizophrenia, risk-taking behavior), after excluding overlapping UK Biobank exposure-outcome pairings). Overlapping exposure-outcome pairings were strictly excluded from the primary analyses. The FDR correction was applied exclusively to the *p*-values derived from these eight independent primary IVW regression models, with an FDR-adjusted *p*-value < 0.05 considered statistically significant.

To distinguish a true shared causal signal from linkage disequilibrium (LD)-driven confounding, we performed Bayesian colocalization analysis using the R package ‘coloc’ [47]. This analysis evaluates five mutually exclusive hypotheses: PP.H0 (no genetic association with either trait), PP.H1 (genetic association with the exposure only), PP.H2 (genetic association with the outcome only), PP.H3 (genetic association with both traits, driven by distinct independent causal variants in LD), and PP.H4 (genetic association with both traits, sharing a single identical underlying causal variant). A posterior probability for H4 (PP.H4) > 0.80 was considered robust evidence of a shared causal variant. Strictly screened lead SNPs from the exposure dataset were selected as index variants, and genomic regions extending ±500 kb around each lead SNP were extracted for colocalization analysis.

In MR analyses, statistical power is governed by the proportion of exposure variance explained by instrumental variables (*R*^2^, S1 and S2 Tables), the case-to-control ratio, and the causal effect size. Following the framework by Brion et al. [18], power is calculated using the non-centrality parameter (NCP) of a non-central chi-square distribution, estimating the probability of detecting true causal associations at a given significance level. Therefore, stronger instruments, larger cohorts with case-to-control ratios close to 1:1, and larger effect sizes yield higher statistical power. In contrast, weak instruments and imbalanced sample ratios hinder the detection of modest effects and increase false-negative risks. Rather than reporting post-hoc power based on observed effect sizes—which is statistically circular as it is a direct function of the *p*-value—we calculated the minimum detectable effect size (MDES) at 80% power for each primary exposure-outcome pair. For binary outcomes, the effective sample size was computed as $N_{eff}\;=\;\text{4}\;\times \;N_{case}\;\times \;N_{control}\;/\;(N_{case}\;+\;N_{control})$. The minimum detectable log-odds ratio was derived by solving $NCP\;=\;R^{\text{2}}\;\times \;N_{eff}\;\times \;\beta^{\text{2}}$ for *β* at the NCP corresponding to 80% power (NCP = 7.85 at α = 0.05).

## Supporting information

S1 Fig MR Analysis (Exposure: ebi-a-GCST90006925 Outcome: ebi-a-GCST003724 (Bipolar Disorder)). (DOCX)

S2 Fig MR Analysis (Exposure: ebi-a-GCST90006925 Outcome: ieu-a-801 (Bipolar Disorder)). (DOCX)

S3 Fig MR Analysis (Exposure: ebi-a-GCST90006925 Outcome: ieu-b-41 (Bipolar Disorder)). (DOCX)

S4 Fig MR Analysis (Exposure: ebi-a-GCST90006925 Outcome: ieu-a-1189 (Obsessive Compulsive Disorder)). (DOCX)

S5 Fig MR Analysis (Exposure: ebi-a-GCST90006925 Outcome: ieu-b-42 (Schizophrenia)). (DOCX)

S6 Fig MR Analysis (Exposure: ebi-a-GCST90006925 Outcome: finn-b-F5_SCHIZO (Schizophrenia)). (DOCX)

S7 Fig MR Analysis (Exposure: ebi-a-GCST90006925 Outcome: finn-b-F5_SCHZPHR (Schizophrenia)). (DOCX)

S8 Fig MR Analysis (Exposure: ebi-a-GCST90006925 Outcome: finn-b-KRA_PSY_SCHIZODEL (Schizophrenia)). (DOCX)

S9 Fig MR Analysis (Exposure: ebi-a-GCST90006925 Outcome: finn-b-KRA_PSY_SCHIZODEL_EXMORE (Schizophrenia)). (DOCX)

S10 Fig MR Analysis (Exposure: ieu-b-4910 Outcome: ukb-d-20552_2 (Addiction)). (DOCX)

S11 Fig MR Analysis (Exposure: ieu-b-4910 Outcome: ukb-d-20415 (Addiction)). (DOCX)

S12 Fig MR Analysis (Exposure: ieu-b-4910 Outcome: ebi-a-GCST003724 (Bipolar Disorder)). (DOCX)

S13 Fig MR Analysis (Exposure: ieu-b-4910 Outcome: ieu-a-801 (Bipolar Disorder)). (DOCX)

S14 Fig MR Analysis (Exposure: ieu-b-4910 Outcome: ieu-b-41 (Bipolar Disorder)). (DOCX)

S15 Fig MR Analysis (Exposure: ieu-b-4910 Outcome: ieu-b-5110 (Bipolar Disorder)). (DOCX)

S16 Fig MR Analysis (Exposure: ieu-b-4910 Outcome: ieu-a-1189 (Obsessive Compulsive Disorder)). (DOCX)

S17 Fig MR Analysis (Exposure: ieu-b-4910 Outcome: ieu-b-5102 (Schizophrenia)). (DOCX)

S18 Fig MR Analysis (Exposure: ieu-b-4910 Outcome: ieu-b-42 (Schizophrenia)). (DOCX)

S19 Fig MR Analysis (Exposure: ieu-b-4910 Outcome: finn-b-F5_SCHIZO (Schizophrenia)). (DOCX)

S20 Fig MR Analysis (Exposure: ieu-b-4910 Outcome: finn-b-F5_SCHZPHR (Schizophrenia)). (DOCX)

S21 Fig MR Analysis (Exposure: ieu-b-4910 Outcome: finn-b-KRA_PSY_SCHIZODEL (Schizophrenia)). (DOCX)

S22 Fig MR Analysis (Exposure: ieu-b-4910 Outcome: finn-b-KRA_PSY_SCHIZODEL_EXMORE (Schizophrenia)). (DOCX)

S23 Fig MR Analysis (Exposure: ieu-b-4910 Outcome: ukb-b-14147 (Risk-taking Behavior)). (DOCX)

S24 Fig MR Analysis (Exposure: ieu-b-4910 Outcome: ebi-a-GCST90013958 (Risk-taking Behavior)). (DOCX)

S1 Table Characteristics of screened SNPs associated with *T. gondii* infection (GWAS data: ebi-a-GCST90006925). (DOCX)

S2 Table Characteristics of screened SNPs associated with *T. gondii* infection (GWAS data: ieu-b-4910). (DOCX)

S3 Table Summary of statistical tests for Meta-analyses (Exposure data: ebi-a-GCST90006925). (DOCX)

S4 Table Summary of statistical tests for Meta-analyses (Exposure data: ieu-b-4910). (DOCX)

S5 Table Summary of sensitivity analysis for MR analyses (Exposure data: ebi-a-GCST90006925). (DOCX)

S6 Table Summary of sensitivity analysis for MR analyses (Exposure data: ieu-b-4910). (DOCX)

S7 Table Summary of reverse two-sample MR analyses using three regression approaches (Outcome data: ebi-a-GCST90006925). (DOCX)

S8 Table Summary of reverse two-sample MR analyses using three regression approaches (Outcome data: ieu-b-4910). (DOCX)

S9 Table Summary on sensitivity analysis for reverse two-sample MR analyses (Outcome data: ebi-a-GCST90006925). (DOCX)

S10 Table Summary on sensitivity analysis for reverse two-sample MR analyses (Outcome data: ieu-b-4910). (DOCX)

S11 Table Results of colocalization analysis. (XLSX)

S12 Table Summary of MR analyses with four regression approaches (IVW, MR-Egger, Weighted Median, and MR-PRESSO). (DOCX)

S1 Dataset Datasets. (DOCX)

S1 STROBE Checklist Checklist of recommended items to address in reports of Mendelian randomization studies. (DOCX)
