# Supplementary material for: Does Toxoplasma gondii infection have a causal impact on human psychopathology? A Mendelian randomization analysis
Source: PLoS Pathog. 2026 Aug 4;22(8):e1014312. doi: 10.1371/journal.ppat.1014312 (PMC13450837; doi:10.1371/journal.ppat.1014312)
Supplement: Supplementary file 8 [file ppat.1014312.s008.docx]

| A. MR REGRESSION   | B. FOREST PLOT FOR EACH SNP’S EFFECT   |
| --- | --- |
| C. FUNNEL PLOT  | D. LEAVE-ONE-OUT FOREST PLOT  |

**S8 Fig.** MR Analysis (Exposure: ebi-a-GCST90006925 Outcome: finn-b-KRA_PSY_SCHIZODEL (Schizophrenia))
