# Supplementary material for: Does Toxoplasma gondii infection have a causal impact on human psychopathology? A Mendelian randomization analysis
Source: PLoS Pathog. 2026 Aug 4;22(8):e1014312. doi: 10.1371/journal.ppat.1014312 (PMC13450837; doi:10.1371/journal.ppat.1014312)
Supplement: Supplementary file 25 [file ppat.1014312.s025.docx]

**S1 Table.** Characteristics of screened SNPs associated with *T. gondii* infection (GWAS data: ebi-a-GCST90006925).

| **SNP** | **Chr.** | **Pos.** | **Effect allele** | **Other allele** | **EAF** | **Beta** | **SE** | ***p*-value** | **R^2^** | ***F*-statistics** |
| --- | --- | --- | --- | --- | --- | --- | --- | --- | --- | --- |
| rs116029235 | 1 | 77090938 | G | A | 0.0239 | 0.0978 | 0.0218 | 7.24E-06 | 0.0023 | 20.1246 |
| rs238080 | 1 | 229475610 | T | C | 0.2687 | 0.0351 | 0.0077 | 4.83E-06 | 0.0024 | 20.8975 |
| rs1059704 | 1 | 206648378 | A | G | 0.7112 | -0.0376 | 0.0077 | 8.96E-07 | 0.0028 | 24.1331 |
| rs78797285 | 2 | 3058103 | C | T | 0.0464 | 0.0733 | 0.0160 | 4.59E-06 | 0.0024 | 20.9964 |
| rs2009627 | 3 | 187600359 | G | A | 0.3634 | -0.0309 | 0.0070 | 9.70E-06 | 0.0022 | 19.5658 |
| rs13105526 | 4 | 189076833 | C | G | 0.8323 | 0.0429 | 0.0090 | 1.94E-06 | 0.0026 | 22.6489 |
| rs13182179 | 5 | 110781665 | T | G | 0.3721 | 0.0311 | 0.0070 | 9.38E-06 | 0.0022 | 19.6290 |
| rs3203960 | 5 | 167891830 | C | T | 0.4146 | -0.0308 | 0.0068 | 6.68E-06 | 0.0023 | 20.2782 |
| rs7727080 | 5 | 144230668 | G | A | 0.1717 | 0.0459 | 0.0089 | 2.18E-07 | 0.0031 | 26.8640 |
| rs56064451 | 6 | 36980701 | G | C | 0.1056 | 0.0510 | 0.0108 | 2.54E-06 | 0.0025 | 22.1346 |
| rs1636613 | 7 | 76066916 | G | A | 0.3237 | 0.0399 | 0.0089 | 7.27E-06 | 0.0023 | 20.1167 |
| rs7795993 | 7 | 157746883 | C | T | 0.3813 | -0.0379 | 0.0085 | 9.01E-06 | 0.0023 | 19.7053 |
| rs12536170 | 7 | 13214540 | A | G | 0.0619 | 0.0607 | 0.0137 | 9.99E-06 | 0.0022 | 19.5085 |
| rs12670088 | 7 | 57404338 | C | G | 0.0267 | 0.0965 | 0.0210 | 4.46E-06 | 0.0024 | 21.0500 |
| rs78496831 | 8 | 143597521 | A | G | 0.0175 | 0.1177 | 0.0254 | 3.72E-06 | 0.0024 | 21.3988 |
| rs10830175 | 10 | 129532506 | G | A | 0.9842 | -0.1188 | 0.0266 | 7.86E-06 | 0.0023 | 19.9677 |
| rs9554177 | 13 | 28430981 | A | G | 0.4080 | -0.0303 | 0.0068 | 9.07E-06 | 0.0022 | 19.6933 |
| rs117643464 | 13 | 101945205 | A | T | 0.0277 | -0.0930 | 0.0206 | 6.22E-06 | 0.0023 | 20.4159 |
| rs147285933 | 15 | 57635954 | T | G | 0.0478 | -0.0694 | 0.0155 | 7.47E-06 | 0.0023 | 20.0651 |
| rs118101855 | 15 | 58283358 | G | A | 0.0108 | 0.1710 | 0.0326 | 1.56E-07 | 0.0031 | 27.5099 |
| rs8044719 | 16 | 56678865 | G | T | 0.8464 | -0.0425 | 0.0093 | 5.30E-06 | 0.0024 | 20.7225 |
| rs72832765 | 17 | 6811380 | G | T | 0.0382 | 0.0875 | 0.0176 | 6.31E-07 | 0.0028 | 24.8104 |
| rs138066328 | 18 | 32005865 | T | C | 0.0176 | 0.1181 | 0.0254 | 3.23E-06 | 0.0025 | 21.6721 |
| rs4356554 | 18 | 5910953 | T | C | 0.0520 | 0.0788 | 0.0152 | 2.02E-07 | 0.0031 | 27.0051 |
| rs111833847 | 19 | 50033838 | T | C | 0.0295 | 0.0881 | 0.0199 | 8.99E-06 | 0.0023 | 19.7112 |
| **Total** |  |  |  |  |  |  |  |  | **0.0617** |  |
