# Supplementary material for: Does Toxoplasma gondii infection have a causal impact on human psychopathology? A Mendelian randomization analysis
Source: PLoS Pathog. 2026 Aug 4;22(8):e1014312. doi: 10.1371/journal.ppat.1014312 (PMC13450837; doi:10.1371/journal.ppat.1014312)
Supplement: Supplementary file 26 [file ppat.1014312.s026.docx]

**S2 Table.** Characteristics of screened SNPs associated with *T. gondii* infection (GWAS data: ieu-b-4910).

| **SNP** | **Chr.** | **Pos.** | **Effect allele** | **Other allele** | **EAF** | **Beta** | **SE** | ***p*-value** | **R^2^** | ***F*-statistics** |
| --- | --- | --- | --- | --- | --- | --- | --- | --- | --- | --- |
| rs143787821 | 1 | 53836391 | A | G | 0.0335 | 0.2558 | 0.0626 | 4.41E-05 | 0.0036 | 18.0984 |
| rs72717048 | 1 | 161500796 | G | C | 0.0809 | -0.2186 | 0.0425 | 2.74E-07 | 0.0034 | 17.3309 |
| rs626502 | 1 | 32387492 | G | T | 0.4312 | 0.1032 | 0.0228 | 6.43E-06 | 0.0036 | 17.9263 |
| rs10788882 | 1 | 48630438 | C | G | 0.3364 | -0.1161 | 0.0236 | 8.81E-07 | 0.0100 | 50.8076 |
| rs10800607 | 1 | 199029511 | C | T | 0.3063 | 0.0990 | 0.0241 | 3.96E-05 | 0.0033 | 16.7087 |
| rs114230280 | 1 | 113465939 | G | A | 0.0526 | -0.2104 | 0.0504 | 3.06E-05 | 0.0039 | 19.6035 |
| rs75827100 | 2 | 129391144 | G | A | 0.0178 | 0.3980 | 0.0896 | 9.14E-06 | 0.0034 | 17.1052 |
| rs11685108 | 2 | 33514753 | G | A | 0.0809 | -0.1673 | 0.0411 | 4.85E-05 | 0.0037 | 18.8393 |
| rs10865311 | 2 | 59451499 | A | G | 0.0279 | 0.3121 | 0.0669 | 3.15E-06 | 0.0036 | 17.9013 |
| rs16866763 | 2 | 8798824 | C | T | 0.0777 | -0.1688 | 0.0408 | 3.56E-05 | 0.0036 | 17.9231 |
| rs565862 | 3 | 187682285 | A | T | 0.1222 | 0.1554 | 0.0338 | 4.50E-06 | 0.0036 | 18.1954 |
| rs77236966 | 3 | 57379507 | A | G | 0.0213 | 0.3380 | 0.0794 | 2.14E-05 | 0.0034 | 17.2988 |
| rs115300833 | 3 | 128475430 | G | A | 0.0337 | -0.2624 | 0.0629 | 3.07E-05 | 0.0033 | 16.4927 |
| rs9809724 | 3 | 142599435 | T | C | 0.1538 | -0.1385 | 0.0323 | 1.80E-05 | 0.0042 | 20.9730 |
| rs12487717 | 3 | 98605007 | A | G | 0.0122 | 0.5351 | 0.1055 | 4.13E-07 | 0.0033 | 16.7972 |
| rs12495401 | 3 | 9875534 | A | G | 0.3047 | -0.1105 | 0.0243 | 5.59E-06 | 0.0036 | 17.8916 |
| rs9828620 | 3 | 2909434 | A | G | 0.3715 | -0.0961 | 0.0236 | 4.74E-05 | 0.0033 | 16.5832 |
| rs9288916 | 3 | 110493206 | T | C | 0.1702 | 0.1252 | 0.0292 | 1.80E-05 | 0.0039 | 19.4813 |
| rs149882294 | 3 | 190333692 | A | G | 0.0266 | -0.3171 | 0.0742 | 1.97E-05 | 0.0045 | 22.5589 |
| rs1595009 | 4 | 74834656 | T | C | 0.1800 | 0.1209 | 0.0294 | 4.02E-05 | 0.0034 | 17.2023 |
| rs3853438 | 4 | 111810048 | A | G | 0.3130 | 0.1065 | 0.0238 | 7.66E-06 | 0.0041 | 20.6658 |
| rs17468228 | 4 | 11308233 | G | A | 0.0282 | -0.2909 | 0.0703 | 3.55E-05 | 0.0040 | 20.3267 |
| rs114164692 | 5 | 2229452 | G | C | 0.0305 | -0.2967 | 0.0694 | 1.95E-05 | 0.0035 | 17.5660 |
| rs151234658 | 5 | 3072687 | A | G | 0.0144 | -0.4204 | 0.0971 | 1.54E-05 | 0.0035 | 17.5414 |
| rs6897188 | 5 | 71022741 | T | C | 0.1640 | -0.1236 | 0.0304 | 4.90E-05 | 0.0036 | 18.3300 |
| rs784581 | 5 | 78136755 | C | T | 0.4676 | 0.0943 | 0.0225 | 2.78E-05 | 0.0040 | 20.2603 |
| rs6886845 | 5 | 163639923 | C | T | 0.0968 | -0.1617 | 0.0377 | 1.83E-05 | 0.0036 | 18.0081 |
| rs116151398 | 5 | 174558253 | G | A | 0.0158 | 0.4101 | 0.0929 | 1.03E-05 | 0.0033 | 16.5438 |
| rs4701774 | 5 | 7213053 | G | C | 0.4807 | 0.1061 | 0.0223 | 1.99E-06 | 0.0040 | 20.1576 |
| rs77899559 | 6 | 158805182 | T | A | 0.0379 | -0.2426 | 0.0584 | 3.33E-05 | 0.0034 | 17.2719 |
| rs34846419 | 6 | 41694773 | A | G | 0.0817 | -0.1708 | 0.0416 | 4.15E-05 | 0.0034 | 17.1911 |
| rs12190037 | 6 | 54406780 | G | A | 0.0125 | 0.4809 | 0.1168 | 3.92E-05 | 0.0035 | 17.6841 |
| rs141379923 | 6 | 98729717 | T | A | 0.0106 | 0.4991 | 0.1145 | 1.33E-05 | 0.0034 | 17.0839 |
| rs1264457 | 6 | 30458064 | A | G | 0.3994 | -0.1019 | 0.0228 | 7.98E-06 | 0.0040 | 20.1132 |
| rs188609827 | 7 | 3086871 | T | C | 0.0293 | -0.2863 | 0.0679 | 2.55E-05 | 0.0034 | 17.2944 |
| rs2436973 | 8 | 103581619 | C | T | 0.3381 | -0.1050 | 0.0245 | 1.80E-05 | 0.0036 | 18.0984 |
| rs2904702 | 8 | 17102238 | C | T | 0.0232 | -0.3177 | 0.0772 | 3.96E-05 | 0.0034 | 17.3309 |
| rs79875680 | 9 | 6928539 | G | T | 0.0277 | 0.2984 | 0.0730 | 4.46E-05 | 0.0036 | 17.9263 |
| rs11597743 | 10 | 72314089 | T | G | 0.4205 | 0.1036 | 0.0230 | 7.08E-06 | 0.0100 | 50.8076 |
| rs11190645 | 10 | 83369717 | G | A | 0.0257 | -0.3173 | 0.0707 | 7.46E-06 | 0.0033 | 16.7087 |
| rs112501919 | 10 | 126926577 | T | C | 0.0381 | -0.2466 | 0.0583 | 2.37E-05 | 0.0039 | 19.6035 |
| rs80343370 | 11 | 114547621 | C | T | 0.0364 | 0.2750 | 0.0646 | 2.14E-05 | 0.0034 | 17.1052 |
| rs117352611 | 11 | 57214639 | T | C | 0.0229 | 0.3181 | 0.0764 | 3.20E-05 | 0.0037 | 18.8393 |
| rs7121919 | 11 | 72496087 | A | G | 0.3751 | -0.0979 | 0.0231 | 2.34E-05 | 0.0036 | 17.9013 |
| rs36020612 | 11 | 123344435 | T | C | 0.1939 | 0.2022 | 0.0284 | 1.20E-12 | 0.0036 | 17.9231 |
| rs7938671 | 11 | 94333958 | C | T | 0.0129 | -0.4118 | 0.1007 | 4.43E-05 | 0.0036 | 18.1954 |
| rs77735964 | 11 | 2075720 | G | C | 0.2024 | 0.1241 | 0.0280 | 9.75E-06 | 0.0034 | 17.2988 |
| rs11837434 | 12 | 6203155 | C | T | 0.0422 | -0.2426 | 0.0586 | 3.60E-05 | 0.0033 | 16.4927 |
| rs2585570 | 13 | 78706693 | C | A | 0.0325 | 0.2619 | 0.0603 | 1.45E-05 | 0.0042 | 20.9730 |
| rs17268038 | 13 | 95687671 | G | T | 0.0462 | 0.2375 | 0.0561 | 2.37E-05 | 0.0033 | 16.7972 |
| rs61973639 | 13 | 93130805 | T | C | 0.0335 | 0.2775 | 0.0655 | 2.34E-05 | 0.0036 | 17.8916 |
| rs9535200 | 13 | 49897226 | C | T | 0.3058 | 0.1037 | 0.0243 | 2.03E-05 | 0.0033 | 16.5832 |
| rs117845304 | 14 | 52609910 | T | C | 0.0257 | -0.3143 | 0.0756 | 3.25E-05 | 0.0039 | 19.4813 |
| rs60385209 | 14 | 70758483 | T | C | 0.1628 | 0.1244 | 0.0306 | 4.96E-05 | 0.0045 | 22.5589 |
| rs2119613 | 15 | 98176438 | A | C | 0.3173 | 0.1111 | 0.0243 | 4.78E-06 | 0.0034 | 17.2023 |
| rs11072116 | 15 | 70050992 | C | G | 0.4460 | -0.0922 | 0.0225 | 4.23E-05 | 0.0041 | 20.6658 |
| rs181678142 | 15 | 30941395 | A | C | 0.0191 | 0.4426 | 0.1046 | 2.38E-05 | 0.0040 | 20.3267 |
| rs34593825 | 16 | 83220867 | A | G | 0.0138 | 0.4119 | 0.1011 | 4.73E-05 | 0.0035 | 17.5660 |
| rs1704480 | 16 | 86294325 | G | A | 0.4384 | 0.1009 | 0.0229 | 1.04E-05 | 0.0035 | 17.5414 |
| rs35337779 | 17 | 1383566 | T | C | 0.0464 | 0.2643 | 0.0556 | 2.10E-06 | 0.0036 | 18.3300 |
| rs141159963 | 17 | 15432440 | C | A | 0.0128 | -0.4745 | 0.1144 | 3.42E-05 | 0.0040 | 20.2603 |
| rs142488694 | 17 | 78444577 | C | G | 0.0151 | -0.4229 | 0.0930 | 5.61E-06 | 0.0036 | 18.0081 |
| rs9912230 | 17 | 54846128 | A | T | 0.0161 | 0.4301 | 0.0954 | 6.69E-06 | 0.0033 | 16.5438 |
| rs12949658 | 17 | 36733252 | T | C | 0.0779 | 0.1790 | 0.0427 | 2.83E-05 | 0.0040 | 20.1576 |
| rs2195045 | 18 | 64520173 | T | A | 0.2454 | -0.1088 | 0.0260 | 2.86E-05 | 0.0034 | 17.2719 |
| rs60409321 | 18 | 35413357 | C | A | 0.0162 | -0.3813 | 0.0890 | 1.90E-05 | 0.0034 | 17.1911 |
| rs73444174 | 18 | 47978162 | G | A | 0.0670 | 0.1982 | 0.0440 | 6.93E-06 | 0.0035 | 17.6841 |
| rs72898051 | 18 | 38997470 | A | G | 0.0710 | 0.2148 | 0.0506 | 2.24E-05 | 0.0034 | 17.0839 |
| rs148354939 | 19 | 13712993 | A | C | 0.0593 | -0.2059 | 0.0506 | 4.83E-05 | 0.0040 | 20.1132 |
| rs4803617 | 19 | 43867658 | A | G | 0.3336 | -0.1137 | 0.0253 | 7.31E-06 | 0.0034 | 17.2944 |
| rs916996 | 19 | 1340499 | G | A | 0.2066 | 0.1147 | 0.0276 | 3.30E-05 | 0.0036 | 18.0984 |
| rs73018932 | 19 | 29085712 | T | C | 0.1494 | 0.1337 | 0.0322 | 3.44E-05 | 0.0034 | 17.3309 |
| rs6101905 | 20 | 38836785 | C | T | 0.1476 | -0.1306 | 0.0310 | 2.66E-05 | 0.0036 | 17.9263 |
| rs11698404 | 20 | 566924 | T | C | 0.4335 | -0.0945 | 0.0229 | 3.64E-05 | 0.0100 | 50.8076 |
| rs62220264 | 21 | 16621303 | C | A | 0.0251 | -0.3082 | 0.0687 | 7.48E-06 | 0.0033 | 16.7087 |
| rs78248870 | 21 | 38526212 | C | G | 0.0796 | -0.1785 | 0.0429 | 3.26E-05 | 0.0039 | 19.6035 |
| **Total** |  |  |  |  |  |  |  |  | **0.2944** |  |
