# Supplementary material for: Does Toxoplasma gondii infection have a causal impact on human psychopathology? A Mendelian randomization analysis
Source: PLoS Pathog. 2026 Aug 4;22(8):e1014312. doi: 10.1371/journal.ppat.1014312 (PMC13450837; doi:10.1371/journal.ppat.1014312)
Supplement: Supplementary file 27 [file ppat.1014312.s027.docx]

**S3 Table.** Summary of statistical tests for Meta-analyses (Exposure data: anti-*T. gondii* IgG Seropositivity (ebi-a-GCST90006925)).

|  | beta | se | z | pval | Confidence Interval | Heterogeneity Test | | I^2^ | tau^2^(se) | Egger’s Test | |
| --- | --- | --- | --- | --- | --- | --- | --- | --- | --- | --- | --- |
|  |  |  |  |  |  | Cochran’s Q(df) | pval |  |  | z | pval |
| ieu-b-42 & finn-b-KRA_PSY_SCHIZODEL_EXMORE | -0.051 | 0.080 | -0.634 | 0.526 | (-0.209, 0.107) | 0.402(1) | 0.526 | 0 | 0(0.029) | 0.882 | 0.348 |
| ieu-b-42 & finn-b-KRA_PSY_SCHIZODEL | -0.046 | 0.078 | -0.588 | 0.557 | (-0.198, 0.107) | 0.346(1) | 0.557 | 0 | 0(0.023) | 0.508 | 0.476 |
| ieu-b-42 & finn-b-F5_SCHZPHR | 0.001 | 0.083 | 0.000 | 1.000 | (-0.163, 0.163) | 0.000(1) | 1.000 | 0 | 0(0.040) | 0.162 | 0.687 |
| ieu-b-42 & finn-b-F5_SCHIZO | -0.049 | 0.078 | -0.627 | 0.531 | (-0.201, 0.104) | 0.393(1) | 0.531 | 0 | 0(0.023) | 0.607 | 0.436 |
