# Supplementary material for: Does Toxoplasma gondii infection have a causal impact on human psychopathology? A Mendelian randomization analysis
Source: PLoS Pathog. 2026 Aug 4;22(8):e1014312. doi: 10.1371/journal.ppat.1014312 (PMC13450837; doi:10.1371/journal.ppat.1014312)
Supplement: Supplementary file 28 [file ppat.1014312.s028.docx]

**S4 Table.** Summary of statistical tests for Meta-analyses (Exposure data: anti-*T. gondii* IgG levels (ieu-b-4910)).

|  | beta | se | z | pval | Confidence Interval | Heterogeneity Test | | I^2^ | tau^2^(se) | Egger’s Test | |
| --- | --- | --- | --- | --- | --- | --- | --- | --- | --- | --- | --- |
|  |  |  |  |  |  | Cochran’s Q(df) | pval |  |  | z | pval |
| ieu-b-42 & finn-b-KRA_PSY_SCHIZODEL_EXMORE | -0.004 | 0.015 | -0.258 | 0.796 | (-0.209, 0.107) | 0.067(1) | 0.796 | 0 | 0(0.001) | 0.063 | 0.802 |
| ieu-b-42 & finn-b-KRA_PSY_SCHIZODEL | -0.003 | 0.014 | -0.227 | 0.820 | (-0.198, 0.107) | 0.052(1) | 0.820 | 0 | 0(0.001) | 0.024 | 0.877 |
| ieu-b-42 & finn-b-F5_SCHZPHR | -0.007 | 0.015 | -0.449 | 0.653 | (-0.163, 0.163) | 0.202(1) | 0.653 | 0 | 0(0.001) | 0.582 | 0.446 |
| ieu-b-42 & finn-b-F5_SCHIZO | -0.003 | 0.014 | -0.222 | 0.825 | (-0.201, 0.104) | 0.049(1) | 0.825 | 0 | 0(0.001) | 0.021 | 0.884 |
| ieu-b-5102 & finn-b-KRA_PSY_SCHIZODEL_EXMORE | 0.003 | 0.012 | 0.286 | 0.775 | (-0.033, 0.025) | 0.082(1) | 0.775 | 0 | 0(0.001) | 0.234 | 0.629 |
| ieu-b-5102 & finn-b-KRA_PSY_SCHIZODEL | 0.003 | 0.012 | 0.296 | 0.767 | (-0.031, 0.025) | 0.088(1) | 0.767 | 0 | 0(0.001) | 0.174 | 0.676 |
| ieu-b-5102 & finn-b-F5_SCHZPHR | 0.002 | 0.012 | 0.148 | 0.882 | (-0.037, 0.023) | 0.022(1) | 0.882 | 0 | 0(0.001) | 0.948 | 0.330 |
| ieu-b-5102 & finn-b-F5_SCHIZO | 0.004 | 0.012 | 0.301 | 0.764 | (-0.031, 0.025) | 0.090(1) | 0.764 | 0 | 0(0.001) | 0.166 | 0.684 |
