# Supplementary material for: Does Toxoplasma gondii infection have a causal impact on human psychopathology? A Mendelian randomization analysis
Source: PLoS Pathog. 2026 Aug 4;22(8):e1014312. doi: 10.1371/journal.ppat.1014312 (PMC13450837; doi:10.1371/journal.ppat.1014312)
Supplement: Supplementary file 29 [file ppat.1014312.s029.docx]

**S5 Table.** Summary of sensitivity analysis for MR analyses (Exposure data: ebi-a-GCST90006925).

|  | **Outcome** | **GWAS ID** | **MR-PRESSO** | | | **Heterogeneity Test** | | **Pleiotropy Test** | | **MR-PRESSO**  **Global Test** | |
| --- | --- | --- | --- | --- | --- | --- | --- | --- | --- | --- | --- |
|  |  |  | **logOR** | **SD** | ***p*-value** | **Cochran’s**  ***Q* (*df*)** | ***Q***  ***p*-value** | **MR-Egger**  **Intercept** | ***p*-value** | ***p*-value** | **Outlier**  **Deleted** |
|  | Bipolar Disorder | ebi-a-GCST003724 | -0.0419 | 0.1266 | 0.7441 | 13.7730(20) | 0.8418 | 0.0227 | 0.1772 | 0.875 | / |
|  |  | ieu-a-801 | -0.4496 | 0.2280 | 0.0961 | 3.3523(6) | 0.7635 | 0.0037 | 0.9320 | 0.745 | / |
|  |  | ieu-b-41 | 0.0076 | 0.1179 | 0.9492 | 22.1840(16) | 0.1374 | -0.0038 | 0.7627 | 0.156 | rs147285933 |
|  |  | ieu-b-5110 | -0.0286 | 0.0693 | 0.6853 | 16.8190(17) | 0.4667 | 0.0041 | 0.5900 | 0.462 | / |
|  | Obsessive Compulsive Disorder | ieu-a-1189 | 0.1742 | 0.2103 | 0.4176 | 15.2518(19) | 0.7065 | 0.0109 | 0.6638 | 0.706 | / |
|  | Schizophrenia | ieu-b-42 | -0.0136 | 0.0898 | 0.8810 | 22.3064(17) | 0.1732 | -0.0104 | 0.2842 | 0.181 | / |
|  |  | finn-b-F5_SCHIZO | -0.1530 | 0.1322 | 0.2623 | 13.1179(18) | 0.7845 | 0.0015 | 0.9303 | 0.781 | / |
|  |  | finn-b-F5_SCHZPHR | 0.0825 | 0.2122 | 0.7020 | 16.5828(18) | 0.5519 | 0.0068 | 0.7832 | 0.515 | / |
|  |  | finn-b-KRA_PSY_SCHIZODEL | -0.1414 | 0.1345 | 0.3071 | 13.5331(18) | 0.7590 | 0.0025 | 0.8842 | 0.775 | / |
|  |  | finn-b-KRA_PSY_SCHIZODEL_EXMORE | -0.2037 | 0.1659 | 0.2354 | 15.0655(18) | 0.6575 | 0.0071 | 0.7256 | 0.651 | / |
