# Supplementary material for: Does Toxoplasma gondii infection have a causal impact on human psychopathology? A Mendelian randomization analysis
Source: PLoS Pathog. 2026 Aug 4;22(8):e1014312. doi: 10.1371/journal.ppat.1014312 (PMC13450837; doi:10.1371/journal.ppat.1014312)
Supplement: Supplementary file 30 [file ppat.1014312.s030.docx]

**S6 Table.** Summary of sensitivity analysis for MR analyses (Exposure data: ieu-b-4910).

|  | **Outcome** | **GWAS ID** | **MR-PRESSO** | | | **Heterogeneity Test** | | **Pleiotropy Test** | | **MR-PRESSO**  **Global Test** | |
| --- | --- | --- | --- | --- | --- | --- | --- | --- | --- | --- | --- |
|  |  |  | **logOR** | **SD** | ***p*-value** | **Cochran’s**  ***Q* (*df*)** | ***Q***  ***p*-value** | **MR-Egger**  **Intercept** | ***p*-value** | ***p*-value** | **Outlier**  **Deleted** |
| Psychiatric Disorders | | | | | | | | | | | |
|  | Addiction | ukb-d-20552_2 | 0.0003 | 0.0005 | 0.5485 | 58.3295(61) | 0.5733 | 0.0002 | 0.3957 | 0.576 | / |
|  |  | ukb-d-20415 | -0.0080 | 0.0178 | 0.6555 | 66.6588(61) | 0.2887 | 0.0085 | 0.1843 | 0.303 | / |
|  | Bipolar Disorder | ebi-a-GCST003724 | -0.0479 | 0.0274 | 0.0857 | 52.8239(62) | 0.7904 | -0.0010 | 0.9261 | 0.809 | / |
|  |  | ieu-a-801 | -0.0505 | 0.0546 | 0.3725 | 6.5329(13) | 0.9246 | -0.0288 | 0.4306 | 0.923 | / |
|  |  | ieu-b-41 | -0.0279 | 0.0204 | 0.1765 | 73.9051(61) | 0.1242 | 0.0022 | 0.7641 | 0.108 | rs60385209 |
|  |  | ieu-b-5110 | -0.0395 | 0.0142 | 0.0072 | 77.3106(61) | 0.0776 | 0.0012 | 0.8224 | 0.077 | / |
|  | Obsessive Compulsive Disorder | ieu-a-1189 | 0.0392 | 0.0545 | 0.4749 | 75.6541(58) | 0.0596 | 0.0025 | 0.8996 | 0.069 | / |
|  | Schizophrenia | ieu-b-5102 | 0.0058 | 0.0130 | 0.6543 | 76.0832(61) | 0.0924 | 0.0017 | 0.7100 | 0.094 | / |
|  |  | ieu-b-42 | -0.0019 | 0.0168 | 0.9120 | 74.5020(60) | 0.0986 | 0.0014 | 0.8159 | 0.094 | rs114230280 rs1264457 |
|  |  | finn-b-F5_SCHIZO | -0.0066 | 0.0233 | 0.7777 | 42.8742(60) | 0.9536 | -0.0066 | 0.5064 | 0.962 | / |
|  |  | finn-b-F5_SCHZPHR | -0.0345 | 0.0346 | 0.3229 | 46.4796(60) | 0.8996 | -0.0127 | 0.3736 | 0.895 | / |
|  |  | finn-b-KRA_PSY_SCHIZODEL | -0.0069 | 0.0243 | 0.7781 | 46.5919(60) | 0.8974 | -0.0057 | 0.5679 | 0.892 | / |
|  |  | finn-b-KRA_PSY_SCHIZODEL_EXMORE | -0.0110 | 0.0317 | 0.7310 | 58.1633(60) | 0.5431 | -0.0120 | 0.3079 | 0.555 | / |
| Risk-taking Behavior | | | | | | | | | | | |
|  | Risk-taking Behavior | ukb-b-14147 | -0.0010 | 0.0013 | 0.4448 | 77.2307(62) | 0.0920 | 0.0000 | 0.9301 | 0.093 | / |
|  |  | ebi-a-GCST90013958 | -0.0075 | 0.0074 | 0.3114 | 74.5109(61) | 0.1146 | 0.0031 | 0.2380 | 0.117 | rs77236966 |
