# Supplementary material for: Does Toxoplasma gondii infection have a causal impact on human psychopathology? A Mendelian randomization analysis
Source: PLoS Pathog. 2026 Aug 4;22(8):e1014312. doi: 10.1371/journal.ppat.1014312 (PMC13450837; doi:10.1371/journal.ppat.1014312)
Supplement: Supplementary file 31 [file ppat.1014312.s031.docx]

**S7 Table.** Summary of reverse two-sample MR analyses using three regression approaches (Outcome data: ebi-a-GCST90006925).

|  | **Exposure/GWAS ID** | **Inverse Variance Weighting** | | | | **MR Egger** | | | **Weighted Median** | | |
| --- | --- | --- | --- | --- | --- | --- | --- | --- | --- | --- | --- |
|  |  | **logOR** | **SE** | ***p*-value** | **OR** | **logOR** | **SE** | ***p*-value** | **logOR** | **SE** | ***p*-value** |
| Bipolar Disorder | | | | | | | | | | | |
|  | ebi-a-GCST003724 | 0.0014 | 0.0087 | 0.8720 | 1.0014 | 0.0178 | 0.0149 | 0.2425 | 0.0079 | 0.0125 | 0.5249 |
|  | ieu-a-801 | -0.0069 | 0.0157 | 0.6620 | 0.9932 | -0.1506 | 0.0699 | 0.0522 | 0.0099 | 0.0204 | 0.6291 |
|  | ieu-b-41 | -0.0124 | 0.0300 | 0.6801 | 0.9877 | 0.0444 | 0.2006 | 0.8298 | -0.0062 | 0.0365 | 0.8660 |
| Obsessive Compulsive Disorder | | | | | | | | | | | |
|  | ieu-a-1189 | -0.0017 | 0.0127 | 0.8944 | 0.9983 | 0.0440 | 0.0389 | 0.2876 | 0.0009 | 0.0163 | 0.9569 |
| Schizophrenia | | | | | | | | | | | |
|  | ieu-b-5102 | 0.0156 | 0.0107 | 0.1458 | 1.0157 | 0.0659 | 0.0408 | 0.1093 | 0.0196 | 0.0156 | 0.2097 |
|  | ieu-b-42 | 0.0076 | 0.0117 | 0.5151 | 1.0077 | 0.0536 | 0.0437 | 0.2244 | 0.0101 | 0.0169 | 0.5487 |
|  | finn-b-F5_SCHIZO | -0.0120 | 0.0145 | 0.4087 | 0.9881 | 0.0004 | 0.0230 | 0.9858 | -0.0206 | 0.0197 | 0.2957 |
|  | finn-b-F5_SCHZPHR | 0.0061 | 0.0183 | 0.7376 | 1.0062 | -0.0976 | 0.1139 | 0.4200 | 0.0092 | 0.0220 | 0.6758 |
|  | finn-b-KRA_PSY_SCHIZODEL | -0.0076 | 0.0147 | 0.6051 | 0.9924 | 0.0011 | 0.0215 | 0.9585 | 0.0041 | 0.0183 | 0.8219 |
|  | finn-b-KRA_PSY_SCHIZODEL_EXMORE | 0.0115 | 0.0180 | 0.5215 | 1.0116 | 0.0101 | 0.0358 | 0.7868 | 0.0060 | 0.0243 | 0.8041 |
