# Supplementary material for: Does Toxoplasma gondii infection have a causal impact on human psychopathology? A Mendelian randomization analysis
Source: PLoS Pathog. 2026 Aug 4;22(8):e1014312. doi: 10.1371/journal.ppat.1014312 (PMC13450837; doi:10.1371/journal.ppat.1014312)
Supplement: Supplementary file 32 [file ppat.1014312.s032.docx]

**S8 Table.** Summary of reverse two-sample MR analyses using three regression approaches (Outcome data: ieu-b-4910).

|  | **Exposure/GWAS ID** | **Inverse Variance Weighting** | | | | **MR Egger** | | | **Weighted Median** | | |
| --- | --- | --- | --- | --- | --- | --- | --- | --- | --- | --- | --- |
|  |  | **logOR** | **SE** | ***p*-value** | **OR** | **logOR** | **SE** | ***p*-value** | **logOR** | **SE** | ***p*-value** |
| Addiction | | | | | | | | | | | |
|  | ukb-d-20552_2 | -0.2274 | 3.1634 | 0.9427 | 0.7966 | -9.5440 | 6.1492 | 0.1415 | 1.9946 | 3.8787 | 0.6071 |
|  | ukb-d-20415 | 0.0646 | 0.1397 | 0.6437 | 1.0668 | -0.2417 | 0.3560 | 0.5224 | -0.0247 | 0.1652 | 0.8813 |
| Bipolar Disorder | | | | | | | | | | | |
|  | ebi-a-GCST003724 | 0.0232 | 0.0363 | 0.5228 | 1.0234 | 0.0388 | 0.0884 | 0.6648 | 0.0685 | 0.0534 | 0.1996 |
|  | ieu-a-801 | 0.0079 | 0.0653 | 0.9034 | 1.0080 | -0.1789 | 0.6900 | 0.8029 | 0.0104 | 0.0882 | 0.9064 |
|  | ieu-b-41 | 0.0844 | 0.0948 | 0.3736 | 1.0880 | -0.3561 | 0.5960 | 0.5690 | 0.0722 | 0.1260 | 0.5665 |
|  | ieu-b-5110 | 0.0249 | 0.0645 | 0.6995 | 1.0252 | 0.3030 | 0.3122 | 0.3376 | 0.0615 | 0.0888 | 0.4882 |
| Obsessive Compulsive Disorder | | | | | | | | | | | |
|  | ieu-a-1189 | -0.0045 | 0.0433 | 0.9177 | 0.9955 | 0.0284 | 0.1317 | 0.8354 | -0.0049 | 0.0592 | 0.9345 |
| Schizophrenia | | | | | | | | | | | |
|  | ebi-a-GCST90018919 | -0.0431 | 0.0505 | 0.3938 | 0.9578 | -0.0672 | 0.0825 | 0.4341 | -0.0355 | 0.0597 | 0.5523 |
|  | ieu-b-5102 | 0.0606 | 0.0368 | 0.0997 | 1.0624 | 0.0677 | 0.1342 | 0.6147 | 0.1109 | 0.0529 | 0.3627 |
|  | ieu-b-42 | 0.0247 | 0.0414 | 0.5511 | 1.0250 | -0.1242 | 0.1487 | 0.4075 | 0.0972 | 0.0603 | 0.1072 |
|  | finn-b-F5_SCHIZO | 0.0568 | 0.0599 | 0.3426 | 1.0585 | -0.0469 | 0.4239 | 0.9156 | 0.0462 | 0.0796 | 0.5614 |
|  | finn-b-F5_SCHZPHR | 0.0568 | 0.0599 | 0.3426 | 1.0585 | -0.0469 | 0.4239 | 0.9156 | 0.0462 | 0.0770 | 0.5482 |
|  | finn-b-KRA_PSY_SCHIZODEL | 0.0321 | 0.0532 | 0.5464 | 1.0326 | -0.0464 | 0.0705 | 0.5271 | 0.0725 | 0.0677 | 0.2843 |
|  | finn-b-KRA_PSY_SCHIZODEL_EXMORE | -0.0673 | 0.0764 | 0.3785 | 0.9349 | -0.2090 | 0.1318 | 0.1638 | 0.0053 | 0.0929 | 0.9547 |
| Risk-taking Behavior | | | | | | | | | | | |
|  | ukb-a-241 | -0.2842 | 1.2009 | 0.8129 | 0.7526 | -0.0702 | 5.9312 | 0.9909 | 1.4702 | 1.6271 | 0.3662 |
|  | ukb-b-14147 | -0.1819 | 0.8982 | 0.8395 | 0.8337 | 4.1604 | 3.5932 | 0.2650 | 0.7363 | 1.2407 | 0.5529 |
|  | ebi-a-GCST90013958 | -0.1441 | 0.2079 | 0.4881 | 0.8658 | -0.1370 | 1.7285 | 0.9384 | -0.1653 | 0.2925 | 0.5720 |
|  | ebi-a-GCST006810 | -2.4289 | 1.6362 | 0.1377 | 0.0881 | 2.5362 | 19.4290 | 0.9012 | -2.5545 | 2.1205 | 0.2283 |
