# Supplementary material for: Does Toxoplasma gondii infection have a causal impact on human psychopathology? A Mendelian randomization analysis
Source: PLoS Pathog. 2026 Aug 4;22(8):e1014312. doi: 10.1371/journal.ppat.1014312 (PMC13450837; doi:10.1371/journal.ppat.1014312)
Supplement: Supplementary file 33 [file ppat.1014312.s033.docx]

**S9 Table.** Summary on sensitivity analysis for reverse two-sample MR analyses (Outcome data: ebi-a-GCST90006925).

|  | **Exposure** | **GWAS ID** | **MR-PRESSO** | | | **Heterogeneity Test** | | **Pleiotropy Test** | | **MR-PRESSO**  **Global Test** | |
| --- | --- | --- | --- | --- | --- | --- | --- | --- | --- | --- | --- |
|  |  |  | **logOR** | **SD** | ***p*-value** | **Cochran’s**  ***Q* (*df*)** | ***Q***  ***p*-value** | **MR-Egger**  **Intercept** | ***p*-value** | ***p*-value** | **Outlier**  **Deleted** |
| Psychiatric Disorders | | | | | | | | | | | |
|  | Addiction | ukb-d-20552_2 | 0.6564 | 0.9867 | 0.5154 | 24.0415(16) | 0.0886 | 0.0014 | 0.8591 | 0.102 | rs10770407 |
|  |  | ukb-d-20415 | -0.0267 | 0.0317 | 0.4217 | 7.2047(9) | 0.6158 | -0.0086 | 0.4741 | 0.607 | / |
|  | Bipolar Disorder | ebi-a-GCST003724 | 0.0014 | 0.0080 | 0.8608 | 23.2014(28) | 0.7229 | -0.0035 | 0.1858 | 0.709 | / |
|  |  | ieu-a-801 | -0.0069 | 0.0157 | 0.6692 | 14.8730(13) | 0.3153 | 0.0197 | 0.0572 | 0.312 | / |
|  |  | ieu-b-41 | -0.0124 | 0.0300 | 0.6888 | 13.5811(10) | 0.1930 | -0.0052 | 0.7810 | 0.185 | / |
|  |  | ieu-b-5110 | -0.0210 | 0.0177 | 0.2416 | 44.6447(43) | 0.4025 | 0.0098 | 0.1469 | 0.402 | / |
|  | Obsessive Compulsive Disorder | ieu-a-1189 | -0.0017 | 0.0127 | 0.8971 | 13.6817(10) | 0.1880 | -0.0120 | 0.2470 | 0.184 | / |
|  | Schizophrenia | ebi-a-GCST90018919 | -0.0107 | 0.0096 | 0.2934 | 5.2322(9) | 0.8136 | 0.0028 | 0.6319 | 0.871 | / |
|  |  | ieu-b-5102 | 0.0156 | 0.0107 | 0.1482 | 148.2731(130) | 0.1303 | -0.0034 | 0.2046 | 0.125 | / |
|  |  | ieu-b-42 | 0.0076 | 0.0117 | 0.5173 | 77.9402(70) | 0.2410 | -0.0039 | 0.2791 | 0.237 | / |
|  |  | finn-b-F5_SCHIZO | -0.0120 | 0.0127 | 0.3626 | 9.1452(12) | 0.6905 | -0.0030 | 0.4994 | 0.704 | / |
|  |  | finn-b-F5_SCHZPHR | 0.0061 | 0.0183 | 0.7462 | 11.0128(8) | 0.2010 | 0.0189 | 0.3868 | 0.183 | / |
|  |  | finn-b-KRA_PSY_SCHIZODEL | -0.0076 | 0.0147 | 0.6145 | 14.9776(12) | 0.2427 | -0.0030 | 0.5752 | 0.281 | / |
|  |  | finn-b-KRA_PSY_SCHIZODEL_EXMORE | 0.0115 | 0.0178 | 0.5360 | 7.8650(8) | 0.4468 | 0.0003 | 0.9629 | 0.462 | / |
| Risk-taking Behavior | | | | | | | | | | | |
|  | Risk-taking Behavior | ukb-a-241 | 0.5407 | 0.3648 | 0.1765 | 9.1738(8) | 0.3278 | -0.0166 | 0.1764 | 0.337 | / |
|  |  | ukb-b-14147 | 0.3897 | 0.2746 | 0.1705 | 26.7211(21) | 0.1803 | -0.0004 | 0.9595 | 0.171 | rs4801000 |
|  |  | ebi-a-GCST90013958 | 0.0701 | 0.0566 | 0.2339 | 20.2241(16) | 0.2103 | -0.0016 | 0.8735 | 0.200 | rs9636107 |
|  |  | ebi-a-GCST006810 | 0.2874 | 0.3705 | 0.4508 | 14.7687(14) | 0.3942 | -0.0036 | 0.7672 | 0.376 | / |
