# Supplementary material for: Does Toxoplasma gondii infection have a causal impact on human psychopathology? A Mendelian randomization analysis
Source: PLoS Pathog. 2026 Aug 4;22(8):e1014312. doi: 10.1371/journal.ppat.1014312 (PMC13450837; doi:10.1371/journal.ppat.1014312)
Supplement: Supplementary file 34 [file ppat.1014312.s034.docx]

**S10 Table.** Summary on sensitivity analysis for reverse two-sample MR analyses (Outcome data: ieu-b-4910).

|  | **Exposure** | **GWAS ID** | **MR-PRESSO** | | | **Heterogeneity Test** | | **Pleiotropy Test** | | **MR-PRESSO**  **Global Test** | |
| --- | --- | --- | --- | --- | --- | --- | --- | --- | --- | --- | --- |
|  |  |  | **logOR** | **SD** | ***p*-value** | **Cochran’s**  ***Q* (*df*)** | ***Q***  ***p*-value** | **MR-Egger**  **Intercept** | ***p*-value** | ***p*-value** | **Outlier**  **Deleted** |
| Psychiatric Disorders | | | | | | | | | | | |
|  | Addiction | ukb-d-20552_2 | -0.2274 | 3.1634 | 0.9436 | 21.6249(16) | 0.1557 | 0.0409 | 0.0236 | 0.1037 | / |
|  |  | ukb-d-20415 | 0.0646 | 0.1397 | 0.6577 | 7.96173(7) | 0.3360 | 0.0409 | 0.0436 | 0.3848 | / |
|  | Bipolar Disorder | ebi-a-GCST003724 | 0.0232 | 0.0327 | 0.4855 | 19.5312(24) | 0.7231 | -0.0026 | 0.0133 | 0.8479 | / |
|  |  | ieu-a-801 | 0.0079 | 0.0653 | 0.9064 | 8.40352(8) | 0.3951 | 0.0235 | 0.0865 | 0.7933 | / |
|  |  | ieu-b-41 | 0.0844 | 0.0887 | 0.3694 | 7.0038(8) | 0.5362 | 0.0418 | 0.0558 | 0.4785 | / |
|  |  | ieu-b-5110 | 0.0249 | 0.0645 | 0.7015 | 49.0526(41) | 0.1815 | -0.0193 | 0.0211 | 0.3680 | / |
|  | Obsessive Compulsive Disorder | ieu-a-1189 | -0.0045 | 0.0433 | 0.9202 | 8.2665(8) | 0.4079 | -0.0092 | 0.0345 | 0.7976 | / |
|  | Schizophrenia | ebi-a-GCST90018919 | -0.0431 | 0.0505 | 0.4120 | 14.6828(11) | 0.1975 | 0.0084 | 0.0220 | 0.7122 | / |
|  |  | ieu-b-5102 | 0.0606 | 0.0368 | 0.1025 | 116.3906(113) | 0.3945 | -0.0005 | 0.0087 | 0.9557 | / |
|  |  | ieu-b-42 | 0.0247 | 0.0406 | 0.5462 | 52.0391(54) | 0.5504 | 0.0130 | 0.0125 | 0.3021 | / |
|  |  | finn-b-F5_SCHIZO | 0.0568 | 0.0599 | 0.3742 | 8.1157(7) | 0.3225 | 0.0195 | 0.0787 | 0.8128 | rs936664 |
|  |  | finn-b-F5_SCHZPHR | 0.0568 | 0.0599 | 0.3742 | 8.1157(7) | 0.3225 | 0.0195 | 0.0787 | 0.8128 | / |
|  |  | finn-b-KRA_PSY_SCHIZODEL | 0.0321 | 0.0532 | 0.5599 | 12.9584(10) | 0.2260 | 0.0328 | 0.0209 | 0.1512 | / |
|  |  | finn-b-KRA_PSY_SCHIZODEL_EXMORE | -0.0673 | 0.0764 | 0.4077 | 9.4819(7) | 0.2199 | 0.0372 | 0.0288 | 0.2440 | / |
| Risk-taking Behavior | | | | | | | | | | | |
|  | Risk-taking Behavior | ukb-a-241 | -0.2842 | 1.2009 | 0.8197 | 7.0319(7) | 0.4256 | -0.0016 | 0.0425 | 0.9717 | / |
|  |  | ukb-b-14147 | -0.1819 | 0.8689 | 0.8368 | 14.9730(16) | 0.5266 | -0.0295 | 0.0237 | 0.2311 | / |
|  |  | ebi-a-GCST90013958 | -0.1441 | 0.2064 | 0.4994 | 10.8399(11) | 0.4568 | -0.0002 | 0.0592 | 0.9968 | / |
|  |  | ebi-a-GCST006810 | -2.4289 | 1.0763 | 0.0648 | 2.5960(6) | 0.8576 | -0.0274 | 0.1069 | 0.8078 | / |
