# Supplementary material for: Does Toxoplasma gondii infection have a causal impact on human psychopathology? A Mendelian randomization analysis
Source: PLoS Pathog. 2026 Aug 4;22(8):e1014312. doi: 10.1371/journal.ppat.1014312 (PMC13450837; doi:10.1371/journal.ppat.1014312)
Supplement: Supplementary file 35 [file ppat.1014312.s036.docx]

**S12 Table. Summary of MR analyses with four regression approaches (IVW, MR-Egger, Weighted Median, and MR-PRESSO).**

|  | **Exposure:** **Anti-*T. gondii* IgG seropositivity (ebi-a-GCST90006925)** | | | | | | | | | | | | |  |  |  |  |  |  |  |  |  |  |  |  |  |  |  |  |  |  |  |  |  |  |
| --- | --- | --- | --- | --- | --- | --- | --- | --- | --- | --- | --- | --- | --- | --- | --- | --- | --- | --- | --- | --- | --- | --- | --- | --- | --- | --- | --- | --- | --- | --- | --- | --- | --- | --- | --- |
|  | **Outcome/GWAS ID** | **Inverse Variance Weighting** | | | | **MR Egger** | | | **Weighted Median** | | | **FDR** | **Statistical**  **Power** |  |  |  |  |  |  |  |  |  |  |  |  |  |  |  |  |  |  |  |  |  |  |
|  |  | **logOR** | **SE** | ***p* value** | **OR** | **logOR** | **SE** | ***p* value** | **logOR** | **SE** | ***p* value** |  |  |  |  |  |  |  |  |  |  |  |  |  |  |  |  |  |  |  |  |  |  |  |  |
| Bipolar Disorder | | | | | | | | | | | |  |  |  |  |  |  |  |  |  |  |  |  |  |  |  |  |  |  |  |  |  |  |  |  |
|  | ebi-a-GCST003724 | -0.0419 | 0.1526 | 0.7836 | 0.9590 | -0.4589 | 0.3344 | 0.1859 | 0.0659 | 0.2062 | 0.7495 | 0.8792 | 1.00 |  |  |  |  |  |  |  |  |  |  |  |  |  |  |  |  |  |  |  |  |  |  |
|  | ieu-a-801 | -0.4496 | 0.3050 | 0.1405 | 0.6379 | -0.5473 | 1.1302 | 0.6487 | -0.5676 | 0.3992 | 0.1551 | - | 1.00 |  |  |  |  |  |  |  |  |  |  |  |  |  |  |  |  |  |  |  |  |  |  |
|  | ieu-b-41 | 0.0076 | 0.1179 | 0.9484 | 1.0077 | 0.0761 | 0.2536 | 0.7683 | 0.0040 | 0.1559 | 0.9796 | - | 0.317 |  |  |  |  |  |  |  |  |  |  |  |  |  |  |  |  |  |  |  |  |  |  |
| Obsessive Compulsive Disorder | | | | | | | | | | | |  |  |  |  | |  | |  | |  | |  | |  | |  | |  | |  | |  | |  |
|  | ieu-a-1189 | 0.1742 | 0.2347 | 0.4578 | 1.1904 | -0.0133 | 0.4849 | 0.9784 | 0.0238 | 0.3446 | 0.9450 | 0.7070 | 1.00 |  |  |  |  |  |  |  |  |  |  |  |  |  |  |  |  |  |  |  |  |  |  |
| Schizophrenia | | | | | | | | | | | |  |  |  |  |  |  |  |  |  |  |  |  |  |  |  |  |  |  |  |  |  |  |  |  |
|  | ieu-b-42 | -0.0136 | 0.0898 | 0.8792 | 0.9864 | 0.1683 | 0.1868 | 0.3811 | -0.0510 | 0.1174 | 0.6639 | 0.8792 | 0.993 |  |  |  |  |  |  |  |  |  |  |  |  |  |  |  |  |  |  |  |  |  |  |
|  | finn-b-F5_SCHIZO | -0.1530 | 0.1548 | 0.3232 | 0.8582 | -0.1802 | 0.3439 | 0.6070 | -0.1785 | 0.2005 | 0.3735 | - | 1.00 |  |  |  |  |  |  |  |  |  |  |  |  |  |  |  |  |  |  |  |  |  |  |
|  | finn-b-F5_SCHZPHR | 0.0825 | 0.2211 | 0.7090 | 1.0860 | -0.0404 | 0.4923 | 0.9355 | 0.1078 | 0.3054 | 0.7241 | - | 1.00 |  |  |  |  |  |  |  |  |  |  |  |  |  |  |  |  |  |  |  |  |  |  |
|  | finn-b-KRA_PSY_  SCHIZODEL | -0.1414 | 0.1551 | 0.3621 | 0.8682 | -0.1869 | 0.3445 | 0.5946 | -0.2171 | 0.2149 | 0.3123 | - | 1.00 |  |  |  |  |  |  |  |  |  |  |  |  |  |  |  |  |  |  |  |  |  |  |
|  | finn-b-KRA_PSY_  SCHIZODEL_EXMORE | -0.2037 | 0.1814 | 0.2614 | 0.8157 | -0.3322 | 0.4032 | 0.4214 | -0.2963 | 0.2530 | 0.2415 | - | 1.00 |  |  |  |  |  |  |  |  |  |  |  |  |  |  |  |  |  |  |  |  |  |  |
| **Exposure: Anti-*T*. *gondii* IgG levels (ieu-b-4910)** | | | | | | | | | | | | | |  |  |  |  |  |  |  |  |  |  |  |  |  |  |  |  |  |  |  |  |  |  |
|  | **Outcome/GWAS ID** | **Inverse Variance Weighting** | | | | **MR Egger** | | | **Weighted Median** | | | **FDR** | **Statistical**  **Power** |  |  |  |  |  |  |  |  |  |  |  |  |  |  |  |  |  |  |  |  |  |  |
|  |  | **logOR** | **SE** | ***p* value** | **OR** | **logOR** | **SE** | ***p* value** | **logOR** | **SE** | ***p* value** |  |  |  |  |  |  |  |  |  |  |  |  |  |  |  |  |  |  |  |  |  |  |  |  |
| Addiction | | | | | | | | | | | |  | |  |  |  |  |  |  |  |  |  |  |  |  |  |  |  |  |  |  |  |  |  |  |
|  | ukb-d-20552_2 | 0.0003 | 0.0005 | 0.5552 | 1.0003 | -0.0006 | 0.0012 | 0.6240 | 0.0008 | 0.0008 | 0.3170 | 0.7070 | 1.00 |  |  |  |  |  |  |  |  |  |  |  |  |  |  |  |  |  |  |  |  |  |  |
|  | ukb-d-20415 | -0.0080 | 0.0178 | 0.6539 | 0.9920 | -0.0543 | 0.0388 | 0.1664 | -0.0134 | 0.0255 | 0.5989 | - | 0.989 |  |  |  |  |  |  |  |  |  |  |  |  |  |  |  |  |  |  |  |  |  |  |
| Bipolar Disorder | | | | | | | | | | | |  |  |  | |  | |  | |  | |  | |  | |  | |  | |  | |  | |  | |
|  | ebi-a-GCST003724 | -0.0479 | 0.0297 | 0.1069 | 0.9533 | -0.0424 | 0.0658 | 0.5219 | -0.0733 | 0.0408 | 0.0724 | - | 1.00 |  |  |  |  |  |  |  |  |  |  |  |  |  |  |  |  |  |  |  |  |  |  |
|  | ieu-a-801 | -0.0505 | 0.0771 | 0.5126 | 0.9508 | 0.1361 | 0.2414 | 0.5832 | -0.0644 | 0.1048 | 0.5386 | - | 1.00 |  |  |  |  |  |  |  |  |  |  |  |  |  |  |  |  |  |  |  |  |  |  |
|  | ieu-b-41 | -0.0279 | 0.0204 | 0.1715 | 0.9725 | -0.0404 | 0.0465 | 0.3882 | -0.0456 | 0.0275 | 0.0973 | - | 1.00 |  |  |  |  |  |  |  |  |  |  |  |  |  |  |  |  |  |  |  |  |  |  |
|  | ieu-b-5110 | -0.0395 | 0.0142 | 0.0054 | 0.9613 | -0.0461 | 0.0328 | 0.1653 | -0.0622 | 0.0190 | 0.0011 | 0.0432 | 1.00 |  |  |  |  |  |  |  |  |  |  |  |  |  |  |  |  |  |  |  |  |  |  |
| Obsessive Compulsive Disorder | | | | | | | | | | | |  |  |  |  |  |  |  |  |  |  |  |  |  |  |  |  |  |  |  |  |  |  |  |  |
|  | ieu-a-1189 | 0.0392 | 0.0545 | 0.4721 | 1.0400 | 0.0246 | 0.1278 | 0.8480 | 0.0514 | 0.0709 | 0.4683 | 0.7070 | 1.00 |  |  |  |  |  |  |  |  |  |  |  |  |  |  |  |  |  |  |  |  |  |  |
| Schizophrenia | | | | | | | | | | | |  |  |  |  |  |  |  |  |  |  |  |  |  |  |  |  |  |  |  |  |  |  |  |  |
|  | ieu-b-42 | -0.0019 | 0.0168 | 0.9116 | 0.9981 | -0.0099 | 0.0383 | 0.7970 | -0.0115 | 0.0227 | 0.6116 | - | 1.00 |  |  |  |  |  |  |  |  |  |  |  |  |  |  |  |  |  |  |  |  |  |  |
|  | ieu-b-5102 | 0.0058 | 0.0130 | 0.6527 | 1.0058 | -0.0043 | 0.0300 | 0.8874 | -0.0024 | 0.0172 | 0.8895 | 0.7459 | 1.00 |  |  |  |  |  |  |  |  |  |  |  |  |  |  |  |  |  |  |  |  |  |  |
|  | finn-b-F5_SCHIZO | -0.0066 | 0.0275 | 0.8106 | 0.9934 | 0.0301 | 0.0614 | 0.6257 | -0.0074 | 0.0399 | 0.8530 | - | 1.00 |  |  |  |  |  |  |  |  |  |  |  |  |  |  |  |  |  |  |  |  |  |  |
|  | finn-b-F5_SCHZPHR | -0.0345 | 0.0393 | 0.3803 | 0.9661 | 0.0359 | 0.0877 | 0.6841 | -0.0400 | 0.0538 | 0.4574 | - | 1.00 |  |  |  |  |  |  |  |  |  |  |  |  |  |  |  |  |  |  |  |  |  |  |
|  | finn-b-KRA_PSY_  SCHIZODEL | -0.0069 | 0.0275 | 0.8030 | 0.9932 | 0.0247 | 0.0615 | 0.6893 | -0.0115 | 0.0399 | 0.7736 | - | 1.00 |  |  |  |  |  |  |  |  |  |  |  |  |  |  |  |  |  |  |  |  |  |  |
|  | finn-b-KRA_PSY_  SCHIZODEL_EXMORE | -0.0110 | 0.0322 | 0.7338 | 0.9891 | 0.0552 | 0.0719 | 0.4460 | -0.0249 | 0.0468 | 0.5948 | - | 1.00 |  |  |  |  |  |  |  |  |  |  |  |  |  |  |  |  |  |  |  |  |  |  |
| Risk-taking Behavior | | | | | | | | | | | |  |  |  |  |  |  |  |  |  |  |  |  |  |  |  |  |  |  |  |  |  |  |  |  |
|  | ukb-b-14147 | -0.0010 | 0.0013 | 0.4419 | 0.9990 | -0.0008 | 0.0029 | 0.7898 | -0.0011 | 0.0017 | 0.5168 | 0.7070 | 1.00 |  |  |  |  |  |  |  |  |  |  |  |  |  |  |  |  |  |  |  |  |  |  |
|  | ebi-a-GCST90013958 | -0.0075 | 0.0074 | 0.3074 | 0.9925 | -0.0249 | 0.0163 | 0.1322 | -0.0100 | 0.0098 | 0.3031 | - | 1.00 |  |  |  |  |  |  |  |  |  |  |  |  |  |  |  |  |  |  |  |  |  |  |
