# Supplementary material for: Does Toxoplasma gondii infection have a causal impact on human psychopathology? A Mendelian randomization analysis
Source: PLoS Pathog. 2026 Aug 4;22(8):e1014312. doi: 10.1371/journal.ppat.1014312 (PMC13450837; doi:10.1371/journal.ppat.1014312)
Supplement: Supplementary file 36 [file ppat.1014312.s037.docx]

**Exposure Datasets**

33204752: Butler-Laporte, G., Kreuzer, D., Nakanishi, T., Harroud, A., Forgetta, V., & Richards, J. B. (2020). Genetic Determinants of Antibody-Mediated Immune Responses to Infectious Diseases Agents: A Genome-Wide and HLA Association Study. Open Forum Infectious Diseases, 7(11), ofaa450. https://doi.org/10.1093/ofid/ofaa450

34804013: Chong, A. H. W., Mitchell, R. E., Hemani, G., Davey Smith, G., Yolken, R. H., Richmond, R. C., & Paternoster, L. (2021). Genetic analyses of common infections in the Avon Longitudinal Study of Parents and Children cohort. Frontiers in Immunology, 12, 727457. https://doi.org/10.3389/fimmu.2021.727457

**Outcome Datasets**

32026800: Davis, K. A. S., Coleman, J. R. I., Adams, M., Allen, N., Breen, G., Cullen, B., Dickens, C., Fox, E., Graham, N., Holliday, J., Howard, L. M., John, A., Lee, W., McCabe, R., McIntosh, A., Pearsall, R., Smith, D. J., Sudlow, C., Ward, J., Zammit, S., & Hotopf, M. (2020). Mental health in UK Biobank - development, implementation and results from an online questionnaire completed by 157 366 participants: A reanalysis. BJPsych Open, 6(2), e18. https://doi.org/10.1192/bjo.2019.100

27329760: Hou, L., Bergen, S. E., Akula, N., et al. (2016). Genome-wide association study of 40,000 individuals identifies two novel loci associated with bipolar disorder. Human Molecular Genetics, 25(15), 3383-3394. https://doi.org/10.1093/hmg/ddw181

21926972: Psychiatric GWAS Consortium Bipolar Disorder Working Group (2011). Large-scale genome-wide association analysis of bipolar disorder identifies a new susceptibility locus near ODZ4. Nature Genetics, 43(10), 977-983. https://doi.org/10.1038/ng.943

31043756: Stahl, E. A., Breen, G., Forstner, A. J., et al. (2019). Genome-wide association study identifies 30 loci associated with bipolar disorder. Nature Genetics, 51(5), 793-803. https://doi.org/10.1038/s41588-019-0397-8

34002096: Mullins, N., Forstner, A. J., O’Connell, K. S., et al. (2021). Genome-wide association study of more than 40,000 bipolar disorder cases provides new insights into the underlying biology. Nature Genetics, 53(6), 817-829. https://doi.org/10.1038/s41588-021-00857-4

28761083: International Obsessive Compulsive Disorder Foundation Genetics Collaborative (IOCDF-GC) and OCD Collaborative Genetics Association Studies (OCGAS) (2018). Revealing the complex genetic architecture of obsessive-compulsive disorder using meta-analysis. Molecular Psychiatry, 23(5), 1181-1188. https://doi.org/10.1038/mp.2017.154

35396580: Trubetskoy, V., Pardinas, A. F., Qi, T., et al. (2022). Mapping genomic loci implicates genes and synaptic biology in schizophrenia. Nature, 604(7906), 502-508. https://doi.org/10.1038/s41586-022-04434-5

25056061: Schizophrenia Working Group of the Psychiatric Genomics Consortium (2014). Biological insights from 108 schizophrenia-associated genetic loci. Nature, 511(7510), 421-427. https://doi.org/10.1038/nature13595

36653562: Kurki, M. I., Karjalainen, J., Palta, P., et al. (2023). FinnGen provides genetic insights from a well-phenotyped isolated population. Nature, 613(7944), 508-518. https://doi.org/10.1038/s41586-022-05473-8

30271922: Clifton, E. A. D., Perry, J. R. B., Imamura, F., et al. (2018). Genome-wide association study for risk taking propensity indicates shared pathways with body mass index. Communications Biology, 1, 36. https://doi.org/10.1038/s42003-018-0042-6
