# Supplementary material for: Does Toxoplasma gondii infection have a causal impact on human psychopathology? A Mendelian randomization analysis
Source: PLoS Pathog. 2026 Aug 4;22(8):e1014312. doi: 10.1371/journal.ppat.1014312 (PMC13450837; doi:10.1371/journal.ppat.1014312)
Supplement: Supplementary file 37 [file ppat.1014312.s038.docx]

**STROBE-MR checklist of recommended items to address in reports of Mendelian randomization studies**^1^ ^2^

| **Item No.** | **Section** | **Checklist item** | **Page No.** | **Relevant text from manuscript** |
| --- | --- | --- | --- | --- |
| 1 | **TITLE and ABSTRACT** | Indicate Mendelian randomization (MR) as the study’s design in the title and/or the abstract if that is a main purpose of the study | 1-2 | Title: "Does Toxoplasma gondii infection have a causal impact on human psychopathology? A Mendelian randomization analysis." Abstract (pp. 1-2) states that two-sample MR analyses were conducted across 18 GWAS datasets. |
|  | **INTRODUCTION** |  |  |  |
| 2 | **Background** | Explain the scientific background and rationale for the reported study.  What is the exposure? Is a potential causal relationship between exposure and outcome plausible?  Justify why MR is a helpful method to address the study question | 4-5 | Introduction (pp. 4-5) describes persistent tissue cysts, possible effects on hormones, neurotransmission, and neuroinflammation, and observational associations with psychiatric and risk-taking outcomes. It explains that MR can mitigate residual confounding and reverse causation if IV assumptions hold. |
| 3 | **Objectives** | State specific objectives clearly, including pre-specified causal hypotheses (if any). State that MR is a method that, under specific assumptions, intends to estimate causal effects | 5-6 | Introduction (pp. 5-6): two-sample MR uses SNPs as IVs to estimate the exposure-outcome association; the stated objective is to assess whether the observed associations with psychiatric disorders and risk-taking behavior are causal. |
|  | **METHODS** |  |  |  |
| 4 | **Study design and data sources** | Present key elements of the study design early in the article. Consider including a table listing sources of data for all phases of the study. For each data source contributing to the analysis, describe the following: |  |  |
|  | a) | Setting: Describe the study design and the underlying population, if possible. Describe the setting, locations, and relevant dates, including periods of recruitment, exposure, follow-up, and data collection, when available. | 19-25 | Study design (p. 19), Data resource (pp. 20-21), Table 1 (p. 21), and phenotype/cohort descriptions (pp. 22-25) describe a two-sample MR study using European-ancestry GWAS summary statistics and its source populations. |
|  | b) | Participants: Give the eligibility criteria, and the sources and methods of selection of participants. Report the sample size, and whether any power or sample size calculations were carried out prior to the main analysis | 20-25; 28; 32 | Data resource and phenotype/cohort sections (pp. 20-25) and Table 1 (p. 21) report source cohorts, sample sizes, ancestry, and case definitions. The power method is described on p. 28 and minimum detectable effect sizes on p. 32. |
|  | c) | Describe measurement, quality control and selection of genetic variants | 27-28 | Instrumental variable selection (pp. 27-28): exposure-specific thresholds, LD clumping with PLINK v1.9, F-statistic screening (F < 10 excluded), PhenoScanner validation, and exclusion of palindromic or allele-mismatched SNPs during harmonization. |
|  | d) | For each exposure, outcome, and other relevant variables, describe methods of assessment and diagnostic criteria for diseases | 21-25 | Table 1 (p. 21) and phenotype/cohort descriptions (pp. 22-25) describe the exposure measures and outcome phenotype definitions, including diagnostic criteria where available. |
|  | e) | Provide details of ethics committee approval and participant informed consent, if relevant | - | Not applicable. |
| 5 | **Assumptions** | Explicitly state the three core IV assumptions for the main analysis (relevance, independence and exclusion restriction) as well assumptions for any additional or sensitivity analysis | 27 | Instrumental variable selection (p. 27) explicitly states the relevance, exclusion-restriction, and independence assumptions for the main MR analysis. |
| 6 | **Statistical methods: main analysis** | Describe statistical methods and statistics used |  |  |
|  | a) | Describe how quantitative variables were handled in the analyses (i.e., scale, units, model) | 20; 22; 27-29; 32 | Seropositivity is a binary exposure and IgG level is continuous (pp. 20, 22). Instrument selection is described on p. 27, MR estimators on pp. 28-29, and the minimum-detectable-effect-size calculation on p. 32. |
|  | b) | Describe how genetic variants were handled in the analyses and, if applicable, how their weights were selected | 27-29 | Variant handling is described on pp. 27-28 (selection, LD clumping, strength, PhenoScanner validation, and harmonization). The IVW, MR-Egger, weighted-median, and MR-PRESSO estimators are described on pp. 28-29. |
|  | c) | Describe the MR estimator (e.g. two-stage least squares, Wald ratio) and related statistics. Detail the included covariates and, in case of two-sample MR, whether the same covariate set was used for adjustment in the two samples | 28-29 | Statistical analysis (pp. 28-29) identifies IVW as the primary estimator and describes MR-Egger, weighted median, and MR-PRESSO. |
|  | d) | Explain how missing data were addressed | - | Not applicable. |
|  | e) | If applicable, indicate how multiple testing was addressed | 31 | Multiple Testing Correction (p. 31): Benjamini-Hochberg FDR correction was applied to eight pre-specified independent primary IVW analyses; FDR-adjusted p < 0.05 was considered statistically significant. |
| 7 | **Assessment of assumptions** | Describe any methods or prior knowledge used to assess the assumptions or justify their validity | 27-32 | Assumptions are stated on p. 27; PhenoScanner validation is reported on p. 28; pleiotropy, heterogeneity, and leave-one-out assessments are described on p. 29; and Bayesian colocalization is described on p. 32. |
| 8 | **Sensitivity analyses and additional analyses** | Describe any sensitivity analyses or additional analyses performed (e.g. comparison of effect estimates from different approaches, independent replication, bias analytic techniques, validation of instruments, simulations) | 28-32 | Additional analyses include MR-Egger, MR-PRESSO, Cochran's Q, leave-one-out and reverse MR (p. 29), cohort-aware meta-analysis (p. 30), FDR correction (p. 31), colocalization and minimum detectable effect size (p. 32), and a priori power calculation (p. 28). |
| 9 | **Software and pre-registration** |  |  |  |
|  | a) | Name statistical software and package(s), including version and settings used | 27-32 | PLINK v1.9 is reported on p. 27; TwoSampleMR, MR-PRESSO, and the power application on p. 28; metafor on p. 30; and coloc on p. 32. |
|  | b) | State whether the study protocol and details were pre-registered (as well as when and where) | - | Not applicable. |
|  | **RESULTS** |  |  |  |
| 10 | **Descriptive data** |  |  |  |
|  | a) | Report the numbers of individuals at each stage of included studies and reasons for exclusion. Consider use of a flow diagram | 7; 21; 27-28 | Table 1 (p. 21) reports source-GWAS sample sizes. Instrument selection and SNP exclusions are described on pp. 27-28. Fig. 1 (p. 7) is a study-design schematic, not a participant flow diagram. |
|  | b) | Report summary statistics for phenotypic exposure(s), outcome(s), and other relevant variables (e.g. means, SDs, proportions) | 21 | Table 1 (p. 21) reports the available sample sizes, case counts, and control counts for exposure and outcome GWAS. Individual-level means and SDs are not reported in this summary-statistics study. |
|  | c) | If the data sources include meta-analyses of previous studies, provide the assessments of heterogeneity across these studies | - | Not applicable. |
|  | d) | For two-sample MR:  i.  Provide justification of the similarity of the genetic variant-exposure associations between the exposure and outcome samples  ii.  Provide information on the number of individuals who overlap between the exposure and outcome studies | 25-27 | Sample Overlap and Bias Assessment (pp. 25-27) evaluates complete UK Biobank overlap, potential PGC3 overlap, and independent ALSPAC, FinnGen, and earlier PGC datasets. |
| 11 | **Main results** |  |  |  |
|  | a) | Report the associations between genetic variant and exposure, and between genetic variant and outcome, preferably on an interpretable scale | 7; Supporting information | Results (p. 7) reports selection of 25 and 76 instruments and refers to S1-S2 Tables. |
|  | b) | Report MR estimates of the relationship between exposure and outcome, and the measures of uncertainty from the MR analysis, on an interpretable scale, such as odds ratio or relative risk per SD difference | 7-9 | Results (pp. 7-9) and Fig. 2 (p. 9) report IVW MR estimates and uncertainty. S12 Table is cited in the Results as the detailed MR-analysis table. |
|  | c) | If relevant, consider translating estimates of relative risk into absolute risk for a meaningful time period | - | Not applicable: the study reports odds-ratio MR estimates and does not estimate absolute risk over a defined time period. |
|  | d) | Consider plots to visualize results (e.g. forest plot, scatterplot of associations between genetic variants and outcome versus between genetic variants and exposure) | 7-12 | Fig. 2 (p. 9) is a forest plot of primary IVW estimates. Results on pp. 7-12 report the supporting MR analyses and leave-one-out results; the detailed plots are cited as S1-S24 Figs. |
| 12 | **Assessment of assumptions** |  |  |  |
|  | a) | Report the assessment of the validity of the assumptions | 7-13; 17 | Results (pp. 7-12) report heterogeneity, pleiotropy, MR-PRESSO, and leave-one-out assessments. Colocalization is reported on p. 13. Discussion (p. 17) reports individual F-statistics above 16 and mean F-statistics above 19. |
|  | b) | Report any additional statistics (e.g., assessments of heterogeneity across genetic variants, such as *I^2^*, Q statistic or E-value) | 12; 17; 32 | MR meta-analysis heterogeneity statistics (Cochran's Q, I2, and tau2) are reported on p. 12; instrument strength on p. 17; and the R2/power framework on p. 32. Detailed SNP characteristics are cited as S1-S2 Tables. |
| 13 | **Sensitivity analyses and additional analyses** |  |  |  |
|  | a) | Report any sensitivity analyses to assess the robustness of the main results to violations of the assumptions | 7-12 | Results (pp. 7-12) report MR-PRESSO outlier testing, MR-Egger intercept tests, heterogeneity testing, and leave-one-out analyses. The meta-analysis robustness assessment is reported on p. 12. S9-S10 are reverse-MR sensitivity tables, not meta-analysis leave-one-out tables. |
|  | b) | Report results from other sensitivity analyses or additional analyses | 12-13; 31-32 | Additional analyses include meta-analysis (p. 12), reverse MR (p. 12), colocalization (p. 13), FDR correction (p. 31), and minimum detectable effect sizes (p. 32). |
|  | c) | Report any assessment of direction of causal relationship (e.g., bidirectional MR) | 12 | Reverse MR analyses are reported on p. 12: no significant effects were detected in the reverse path. S7-S10 Tables are cited for detailed reverse-MR results and sensitivity analyses. |
|  | d) | When relevant, report and compare with estimates from non-MR analyses | 4-5; 15 | Discussion (p. 15) compares MR confidence bounds with effect sizes reported in observational seroepidemiology. Introduction (pp. 4-5) summarizes the relevant non-MR observational evidence. The MR meta-analysis is not a non-MR comparison. |
|  | e) | Consider additional plots to visualize results (e.g., leave-one-out analyses) | 9-12 | Fig. 2 (p. 9) presents primary estimates. Results (pp. 10-12) report leave-one-out analyses; detailed scatter, funnel, forest, and leave-one-out plots are cited as S1-S24 Figs. |
|  | **DISCUSSION** |  |  |  |
| 14 | **Key results** | Summarize key results with reference to study objectives | 14-16 | Discussion (pp. 14-16) summarizes the null findings across the studied outcomes, the confidence bounds for schizophrenia and bipolar disorder, and the likely-artifactual PGC3 bipolar result. |
| 15 | **Limitations** | Discuss limitations of the study, taking into account the validity of the IV assumptions, other sources of potential bias, and imprecision. Discuss both direction and magnitude of any potential bias and any efforts to address them | 17-18 | Limitations (pp. 17-18) addresses European-only analyses, relaxed instrument-selection thresholds and possible weak-instrument/winner's-curse bias, residual pleiotropy, sample overlap, and exposure-measure limitations. |
| 16 | **Interpretation** |  |  |  |
|  | a) | Meaning: Give a cautious overall interpretation of results in the context of their limitations and in comparison with other studies | 14-18 | Discussion (pp. 14-18) gives a cautious interpretation based on confidence bounds, instrument limitations, sensitivity analyses, colocalization, and comparison with observational evidence. |
|  | b) | Mechanism: Discuss underlying biological mechanisms that could drive a potential causal relationship between the investigated exposure and the outcome, and whether the gene-environment equivalence assumption is reasonable. Use causal language carefully, clarifying that IV estimates may provide causal effects only under certain assumptions | 5; 15 | Introduction (p. 5) notes that causal inference requires IV assumptions. Discussion (p. 15) distinguishes binary seropositivity from continuous IgG levels and cautions that IgG instruments may reflect immune-response intensity. |
|  | c) | Clinical relevance: Discuss whether the results have clinical or public policy relevance, and to what extent they inform effect sizes of possible interventions | 15; 19 | Discussion (pp. 15 and 19) constrains the size of any detectable causal effect and gives clinical context. |
| 17 | **Generalizability** | Discuss the generalizability of the study results (a) to other populations, (b) across other exposure periods/timings, and (c) across other levels of exposure | 17-18 | Limitations and future directions (pp. 17-18) discuss restriction to European populations, infection timing/type and exposure heterogeneity, seropositive-cohort analyses, and the need for multi-ancestry studies. |
|  | **OTHER INFORMATION** |  |  |  |
| 18 | **Funding** | Describe sources of funding and the role of funders in the present study and, if applicable, sources of funding for the databases and original study or studies on which the present study is based | - | Not applicable. |
| 19 | **Data and data sharing** | Provide the data used to perform all analyses or report where and how the data can be accessed, and reference these sources in the article. Provide the statistical code needed to reproduce the results in the article, or report whether the code is publicly accessible and if so, where | 37 | Data availability statement (p. 37) provides IEU OpenGWAS access and Table 1 reference numbers. |
| 20 | **Conflicts of Interest** | All authors should declare all potential conflicts of interest | - | Reported in the publication. |

This checklist is copyrighted by the Equator Network under the Creative Commons Attribution 3.0 Unported (CC BY 3.0) license.

1.	Skrivankova VW, Richmond RC, Woolf BAR, Yarmolinsky J, Davies NM, Swanson SA, et al. Strengthening the Reporting of Observational Studies in Epidemiology using Mendelian Randomization (STROBE-MR) Statement. JAMA. 2021;326(16):1614-1621

2.	Skrivankova VW, Richmond RC, Woolf BAR, Davies NM, Swanson SA, VanderWeele TJ, et al. Strengthening the Reporting of Observational Studies in Epidemiology using Mendelian Randomisation (STROBE-MR): Explanation and Elaboration. BMJ. 2021;375:n2233.
